# DNA methylation-mediated repression of microRNA-410 promotes the growth of human glioma cells and triggers cell apoptosis through its interaction with STAT3

**DOI:** 10.1038/s41598-024-51976-x

**Published:** 2024-01-18

**Authors:** Zhang Wenfu, Luo Bin, Rao Binchan, Luo Jingling, Wang Zhenchang, Wan Zhengdi, Yang Lei

**Affiliations:** 1 The First Affiliated Hospital, Guangxi Medical University of Chinese Medicine, Nanning, Guangxi China; 2 Guangxi Medical University of Chinese Medicine, Nanning, Guangxi China; 3 Guangxi Key Laboratory of Integrated Traditional Chinese and Western Medicine and Transformational Medicine for High Incidence Infectious Diseases, Nanning, Guangxi China; 4 Baiyun Hospital of The First Affiliated Hospital of Guangzhou University of Chinese Medicine, Guangzhou, 510470 China; 5 Guizhou Medical University, Guiyang, 550004 Guizhou China

**Keywords:** Cancer, Immunology

## Abstract

This study's purpose was to confirm the observed underexpression of miRNA-410 in glioma tissues and several glioma cells by Quantitative RT-PCR. Our findings suggest that epigenetic alterations occurring at the promoter region of miR-410 may be responsible for the reduced expression of miR-410 in glioma. The occurrence of DNA methylation in the miR-410 promoter was verified to be more prevalent through glioma tissues contrasted to adjacent non-tumor brain tissues through the utilization of methylation-specific PCR and CpG bisulfite sequencing sites in the miR-410 promoter region. Accordantly, miR-410 expression in glioma cell lines was observed to be significantly lesser in comparison to that of the human fetal glial cell line. In addition, it was demonstrated through gain- and loss-of-function investigations that miR-410 exerts significant regulation over cell growth, cell cycle development, and glioma cell apoptosis. The findings of the Luciferase reporter assay and western blot analysis indicate that miR-410 has a direct effect on the 3’-UTR of signal transducer and activator of transcription 3 (STAT3), thereby inhibiting its expression within glioma cells. Besides, our clinical investigation indicates a negative association between miR-410 expression and STAT3 within the glioma tissues of humans. In aggregate, the data provided in this investigation indicates that miR-410 is subjected to underexpression via DNA methylation. Furthermore, it has been observed to perform its function as a tumor suppressor in glioma cells through direct targeting of STAT3. The previously mentioned results could potentially have significant implications for the advancement of a new therapeutic approach for treating glioma.

## Introduction

Glioma is well known as a most frequent malignant tumor affecting the central nervous system, distinguished by a notable level of aggressiveness and a generally unfavorable prognosis^1^. Glioma is commonly classified into four pathological grades (I to IV) in accordance with the clinical and pathological characteristics of malignancy^2^, in accordance with the World Health Organization (WHO) classification^3^. However considerable advances in diagnosis and approximately systemic therapies contributing to the management of glioma, the final prognosis for individuals with glioma, particularly those with high-grade glioma, is unfavorable, as evidenced by a median life expectancy of roughly 15 months and a 5-year survival rate of only 4–5%^4^. Hence, it is crucial to investigate the precise molecular mechanisms characterizing glioma for developing more effective therapeutic approaches^5^.

MicroRNAs (miRNAs) are a conserved family of endogenous, non-coding, single-stranded small (19–22 nucleotides) RNAs that exert a negative modulation on specific target genes expression via either the process of causing the target messenger RNA (mRNA) to be degraded or through the process of inhibiting the translation of mRNA. In previous years, numerous miRNAs were found to be dysregulated in various forms of cancer. Growing evidence has confirmed that miRNAs, acting as either oncogenes or tumor suppressor genes, may exert significant influence on various tumorigenic pathways, including cellular development, proliferation, differentiation, angiogenesis, and apoptosis^6,7^. Therefore, miRNAs possess the ability to serve as valuable diagnostic biomarkers and therapeutic targets for malignancy. Recent studies have suggested that miR-410, located in chromosome 14q32.31, is frequently deregulated and acts as an onco-suppressor in many cancer forms, such as osteosarcoma, breast cancer, liver, and pancreatic cancer^8,9^. A previous study has indicated that there is a reduction in the expression of miR-410 is significantly linked to the disease-free survival of non-MYCN-amplified favorable neuroblastoma. Nevertheless, the precise function of miR-410 in the progression of glioma as well as the underlying mechanisms still remains to be fully understood.

The epigenetic modulation of gene expression is largely promoted by DNA methylation, which is facilitated by DNA methyltransferases (DNMTs). This process primarily occurs at CpG islands situated in the promoter region of genes. Increasing evidence shows DNA methylation can lead to the transcriptional suppression of genes that encode miRNAs and tumor suppressor genes, which may be a possible mechanism in carcinogenesis. It is known that a proportion of around 10% of miRNAs are subject to epigenetic regulation via DNA methylation in the regulatory regions of miRNA'5. In recent studies, it has been confirmed that miRNAs exhibit suppressed expression due to DNA methylation in various cancer types. However, as far as we know, DNA methylation markers in glioma development remain incomplete.

Throughout the current investigation, we sought to confirm the miR-410 underexpression and its potential biological function in glioma specimens and cells. The data presented indicates that the miR-410 expression in glioma cells is subject to epigenetic regulation. In vivo and vitro investigations, both related to gain- and loss-of-function, reveal that restoration of miR-410 expression suppresses glioma cell growth and prompts cell apoptosis, particularly through directly targeting STAT3.

## Materials and methods

### The clinical glioma tissues and cell lines

Twenty pairs of clinical glioma tissues (including 10 cases of glioma tissues at stage I ~ II, 10 cases of glioma tissues at stage III ~ IV) and their respective adjacent normal tissues were acquired subsequent to a thorough review and endorsement by The First Affiliated Hospital of Guangxi Medical University of Chinese Medicine, Nanning, China. Prior to surgery, none of the individuals participating in this study underwent chemotherapy or radiation therapy. Additionally, all patients provided informed consent. Gliomas with pathologic grades I–II were categorized as low-grade ones (well-differentiated gliomas), whereas gliomas with pathologic grades III–IV were categorized as high-grade ones (poorly differentiated gliomas). Following resection, the tissue samples were promptly subjected to flash-frozen in liquid nitrogen. The present study utilized a total of six distinct cell lines, comprising five human glioma cell lines U251(Bio-73178, ATCC), U87(Bio-105821, ATCC), U373(Bio-133633, ATCC), SHG-44(Bio-73148, ATCC), T98G(Bio-69195, ATCC), and one primary human fetal glial cell line (HEB, Bio-105863, ATCC), which have been purchased from the American Type Culture Collection (ATCC, USA). The cells underwent cultivation and sustained in Dul-becco's Modified Eagle Medium (DMEM, Bio-74080, biobw, China), which was enriched with 10% FBS(C0226, beyotime, China). In accordance with ethical guidelines, all experiments involving human subjects in this study were approved by the Ethics Committee of The First Affiliated Hospital of Guangxi university of Chinese Medicine(GXZYYSYJS2020-014–01). Written informed consent was obtained from all participants prior to their inclusion in the study. The study protocol conformed to the ethical standards set forth in the Declaration of Helsinki.

### RNA extraction and quantitative real-time PCR(qRT-PCR)

The isolation of total RNA from glioma tissues and cell lines was conducted utilizing Trizol reagent (15,596,026, Invitrogen, CA, USA) in accordance with the manufacturer's guidelines. The TaqMan miRNA Assay (4,351,372, Applied Biosystems, CA, USA) was employed to investigate miRNA expression. The normalization of miR-410 expression levels was achieved through the utilization of small nuclear RNA U6 expression. The normalization of STAT3 mRNA expression was performed utilizing β-actin. The primer sequences are provided in the supplementary materials(S.Table 1). The qRT-PCR was conducted utilizing ABI 7900 real-time PCR system (Applied Biosystems, USA). All reactions were conducted in three separate runs. The study employed the 2△△Ct approach to assessing the relative quantitative analysis of gene expression for the purpose of determining miR-410 and STAT3 expression levels. The primers utilized in the qRT-PCR analysis were procured from Sangon Co (Shanghai, China).

### Lentivirus infection, 5-Aza-dC treatment, and transfection

The lentiviruses utilized in this investigation consisted of pre-miR-410 (Lv-miR-410), miR-410 antisense (Lv-anti-miR-410), and negative control (Lv-NC). Additionally, miR-410 mimics, miR-410-inhibitor (Lv-anti-miR-410), and their respective scramble mimics (miR-NC), small interfering RNAs (siRNA) targeting human STAT3 mRNA (siR-STAT3) and negative control siRNA (siR-NC) were all obtained from (GenePharma, Shanghai, China). To conduct a stable transfection, U87, and SHG-44 cells were cultivated up to 70% of the plates. Subsequently, they supplemented with a concentration of 2.0 × 10^9^ Pfu/well Lv-miR-410, Lv-anti-miR-410 or negative control lentivirus, selected in 1.5 mg/mL puromycin (Sigma-Aldrich, USA) to generate cell lines U87-Lv-miR-410, U87-Lv-anti-miR-410, U87-Lv-miR-NC, SHG-44-Lv-miR-410, SHG-Lv-anti-miR-410, and SHG-44-Lv-miR-NC. The expression levels of miR-410 were detected using qRT-PCR following a 5-day infection. For the demethylation study, U87 or SHG-44 cells were seeded before in 6 plates, then supplemented with 5-aza-2’-deoxycytidine (5-Aza-dC, Sigma-Aldrich, USA) at 50 μM, 100 μM and fixed for a duration of 72 h. The culture medium, which consisted of 5-Aza-dC, was refreshed on a daily basis. For transfection assays, U87 and SHG-44 cell lines have been transfected with miRNA mimics, miRNA inhibitors, or siRNA through Liopfectamine2000 (11,668,019, Invitrogen, CA, USA) based on the manufacturer's guidelines.

### DNA bisulfite modification and bisulfite-sequencing PCR

The genomic DNA was extracted from tissues or cells utilizing the DNA Extraction Mini Kit (YDP-304, TIANGEN Biotech, Beijing, China) in accordance with the manufacturer's guidelines. A quantity of 1ug of genomic DNA underwent bisulfite treatment utilizing the E.Z.N.ATM Tissue DNA Kit (D3396-01, OMEGA, USA) based on the manufacturer's guidelines. The process of designing PCR primers for the promoter region of the miR-410 gene using bisulfite conversion was conducted utilizing various online tools: MethyPrimer (http://www.urogene.org/cgibin/methprimer/methprimer.cgi)^10^. Bisulfite-sequencing PCR (BSP) for miR-410 was performed in accordance with the subsequent primers(S.Table 1), which have the ability to amplify 347-bp products under specific conditions at a temperature of 95℃ for a period of 10 min, subsequent by 40 cycles of 95℃ for 30 s, 57℃for 30 s, and 72℃for 30 s. The promoter region of miR-410 was found to contain 15 CpG sites in the BSP products. The PCR products were separated with the Gel Extraction Kit (OMEGA, USA) and subsequently cloned into the TA-cloning vectors (Takara, Dalian, China). Sangon Co (Shanghai, China) randomly selected and sequenced six positive clones from each sample.

### Methylation-specific PCR (MSP)

The design of Methylation-specific PCR (MSP) primers targeting the promoter region of the miR-410 gene was performed utilizing online tools: http://www.urogene.org/cgi-bin/methprimer/methprimer.cgi^10^. The primers used for Methylation-specific PCR (MSP) were designed to detect both methylated (M) and unmethylated (U) alleles of the miR-410 promoter(S.Table 1). The MSP primers were initially assessed for their ability to avoid amplification of any DNA that had not undergone bisulfite treatment. The PCR products were subsequently subjected to analysis using a 2.5% agarose gel. In the MSP, samples exhibiting a higher band intensity compared to the negative control were classified as methylated, while samples lacking a visible PCR product were classified as unmethylated.

### Cell proliferative and colony formation assays

The 3-(4, 5-Dimethyl-2-thiazolyl) 22, 5-diphenyl-2-H-tetrazoliμM bromide (MTT) assay was utilized to detect cell proliferation activity. Following a few days of culturing, 10 µl of MTT was introduced to each group well of cells and subsequently underwent incubation for a duration of 1 h within the cell incubator. The determination of optical density (OD) was conducted using a microplate reader with a wavelength of 490 nm. The colony formation assay involved the cultivation of 500 cells per well three times within 6-well plates, and the number of viable cell colonies was identified after a two-week of inoculation. The cellular colonies underwent fixation using 4% paraformaldehyde solution and were subsequently subjected to staining with a 0.5% crystal violet solution for a duration of 30 min. The quantification of colonies exceeding a diameter of 1.5 mm was performed.

### Flow cytometry analysis

The glioma cells have been collected through the process of trypsinization, followed by a wash in ice-cold PBS and subsequently fixed in a solution of 80% ice-cold ethanol in PBS. After fixation, the cells underwent resuspension in cold PBS to a concentration of 5 × 10^5^ cells, after which it was incubated with RNase (10,109,134,001, Sigma-Aldrich, USA) at a temperature of 37 °C for a period of 30 min. To detect the distribution of cell cycles, the cells underwent staining with 20 mg/ml propidium iodide (PI) for 20 min at room temperature. The Annexin V-FITC apoptosis detection kit (88–8102-72, eBioscience, USA) was utilized to label the cells for the purpose of conducting cell apoptosis assays in accordance with the manufacturer's instructions. The flow cytometer (Beckman-Coulter, USA) was utilized to quantify the cell cycle profiles and cell apoptosis rates.

### Dual-luciferase reporter assay

The dual-luciferase reporter assay involved the amplification of the wild-type STAT3-3’-UTR, which contained the predicted binding sites of miR-410. Additionally, a mutant version was constructed through binding site mutagenesis (Fig. 4a). Afterwards, we cloned them into the Sal1/Xho1 site of the pmirGLO vector (GeneSript, Nanjing, China) downstream of the luciferase gene to generate pmirGLO-STAT3-3’-UTR-WT and pmirGLO-STAT3-3’-UTR-Mut vectors. Prior to transfection, U87 and SHG-44 cells underwent inoculation in 96-well plates (5 × 10^3^ cells/well). Subsequently, we employed Lipofectamine 2000 (11,668,019, Invitrogen, CA, USA) to co-transfect 50 or 100 ng of the reporter vector (the pmirGLO-STAT3-3’-UTR vector or the pmirGLO-STAT3-3’-UTR-Mut vector) into the cells. Following a 48-h transfection period, cellular samples were obtained and underwent luciferase activity analysis utilizing the dual-luciferase reporter assay system (Promega, Fitchburg, USA) based on the manufacturer's guidelines.

### Western blot

The glioma cells underwent lysis in RIPA buffer introduced with phenylmethanesulfonyl fluoride (PMSF, ST2573, Beyotime, Beijing, China), and the protein concentration was estimated utilizing a BCA kit (P0009, Beyotime, Beijing, China). Cell lysates underwent separation by 10% SDS-PAGE and transferred onto PVDF membranes (FFP19, Millipore, Billerica, MA) in equal amounts of (60 μg). Following incubation in 5% nonfat milk dissolved in TBS, including 0.1% Tween-20 for a duration of 1 h, The PVDF membranes were subjected to an overnight incubation utilizing primary antibodies(dilution ratio 1:1000) at a temperature of 4℃, followed by subsequent incubation with secondary antibodies(dilution ratio 1:1000) for a duration of 1 h. The visualization of blots was achieved through the utilization of an ECL detection system (P0018S, Beyotime, Beijing, China). The ImageJ software was utilized to quantify the gray intensities of the protein bands. The antibodies utilized were as follows: The rabbit anti-STAT3 antibody (ab68153, Abcam, Cambridge, UK) and the rabbit anti-GAPDH antibody (ab9485, Abcam, Cambridge, UK).

### Xenografted tumor model

A total of fifteen female nude mice, aged four weeks, were procured from the Guangxi Experimental Animal Center (Nanning, China). The animal experiments were granted approval from the Guangxi Medical University Ethics Committee and Guangxi Medical University of Chinese Medicine Ethics Committee. In general, we divided the nude mice into three groups, 2 × 10^6^ U87-Lv-miR-NC, U87-Lv-miR-410 or U87-Lv-anti-miR-410 glioma cells suspended in PBS underwent subcutaneous injection into the left flanks of each group nude mice to establish the xenograft. Tumor volume (mm^3^) was estimated based on the following: volume = (length × width^2^ × 0.5). All mice were sacrificed 42 days post-injection, the tumors underwent excision, and their mass was quantified. After measuring the sizes, the tumors were processed and submitted to Immunohistochemistry analysis. All methods involving animals in this study were performed in strict accordance with the relevant guidelines and regulations. Ethical approval for experiments involving humans was obtained from Ethics Committee of Guangxi university of Chinese Medicine(DW20230630-031). All efforts were made to minimize suffering and ensure the welfare of animals throughout the experiments.

### Immunohistochemistry analysis

The tumors had been fixed in formalin and subsequently incorporated into paraffin. Sections with a thickness of four micrometers were prepared and incubated with a 3% hydrogen peroxide solution for the purpose of suppressing endogenous peroxidase activities. Heat-mediated antigen retrieval was conducted using citrate buffer (pH 6.0). Before incubation with the primary antibody, the slides had been inhibited with goat serum at a concentration of 10%. Prepare dilution solutions at the following concentrations: 1:50, 1:100, 1:200, and 1:500. Subsequently, the specimens were subjected to incubation with antibodies against proliferating cell nuclear antigen (PCNA; M00125-3, Boster, Wuhan, China) or STAT3 (ab202876, Abcam, Cambridge, UK) for an extended duration of time, specifically overnight, within a container that was maintained at a temperature of 4 ℃ and had a humidified environment. The application of immunohistochemical staining was carried out utilizing the 3–3’-diaminobenzidine (DAB) and subsequently subjected to counter-staining with hematoxylin. The immunohistochemistry outcomes were categorized into three groups and evaluated based on the mean staining intensity and surface area. Furthermore, frozen sections of the transplanted tumor tissues were evaluated via TdT-mediated dUTP nick end-labeling (TUNEL) assay using the One Step TUNEL Apoptosis Kit (C1086, Beyotime, Beijing, China). The present study involved the placement of frozen sections of tumor tissue suspensions onto glass slides coated with Poly-L-lysine. The samples were subsequently fixed, permeabilized, and subjected to incubation with the TUNEL reaction mixture at 4˚C overnight, based on the manufacturer's guidelines.

### Anesthesia and euthansia

Anesthesia: For experiments involving animals, we utilized isoflurane inhalation anesthesia. Isoflurane was administered via a precision vaporizer at a concentration of 2% in medical oxygen. Animals were placed in an induction chamber until loss of consciousness was achieved, and then they were transferred to an anesthesia maintenance chamber for the duration of the procedure. During anesthesia, vital signs such as heart rate, respiratory rate, and oxygen saturation were continuously monitored to ensure the animals' well-being. Anesthesia was maintained at a surgical plane throughout the procedures, and animals were allowed to recover in a controlled environment with supplementary oxygen as needed.

Euthanasia: Euthanasia of animals in our study was carried out in strict accordance with ethical guidelines. We employed the intraperitoneal injection of pentobarbital at a dose of 200 mg/kg. Euthanasia was performed by trained personnel following established protocols. The criteria for determining euthanasia endpoints include dabsence of reflexes, and cessation of respiration. Following euthanasia, appropriate post-euthanasia procedures, were conducted as required by our experimental protocols and ethical regulations.

### Ethics statement

We declare that this study was conducted in compliance with the ARRIVE (Animal Research: Reporting of In Vivo Experiments) guidelines, adhering to its core principles and guidelines. We have followed the necessary steps outlined in the ARRIVE guidelines to minimize suffering and ensure appropriate care and welfare for the experimental animals. We have provided detailed descriptions of the methods and procedures of the animal experiments, and have reported relevant statistical analyses and data comprehensively. The method of this study has been approved by the Ethics Committee of The First Affiliated Hospital of Guangxi university of Chinese Medicine, and the animal experiments were approved by the Ethics Committee of Guangxi university of Chinese Medicine(DW20230630-031).

### Statistical analysis

The qRT-PCR assays were conducted in triplicate, and the experimental procedure was conducted three times. All data are shown as the mean ± standard deviation (mean ± SD) of three or more independent investigations, and the variances were deemed statistically significant at *P* < 0.05 as determined by the Student's t-test. The association between the expression of miR-410 and STAT3 was investigated by Spearman's correlation Analysis. The statistical analyses were conducted utilizing SPSS16.0 (Chicago, IL, USA).

## Results

### The expression of miR-410 was underexpressed in glioma

**Figure 1:**
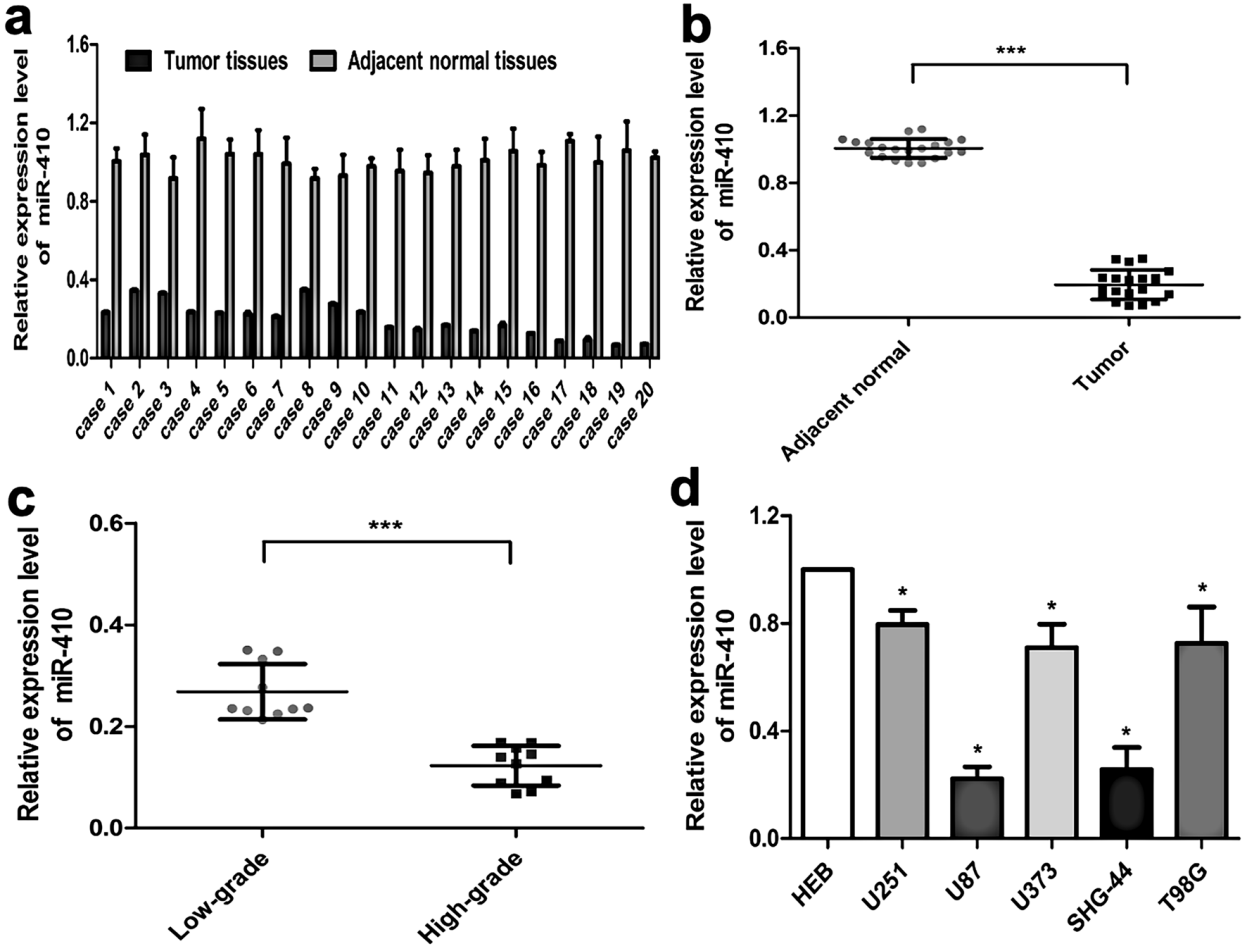
MiR-410 is underexpressed within glioma tissues. (**a, b)** The expression levels of miR-410 were observed in 20 pairs of glioma tissues and their matched adjacent normal brain tissues by qRT-PCR(n = 20). (**c**) qRT-PCR analysis of miR-410 in glioma tissues with various stages(n = 10). (**d**) The relative expression levels of miR-410 in five glioma cell lines (U251, U87, U373, SHG-44, T98G) and normal neuronal cell line HEB. The data presented in this study are expressed as mean ± SD of three independent experiments. all data underwent normalization to U6.*P < 0.05,***P < 0.001 versus the control(n = 6).

In order to evaluate the expression of miR-410 in glioma, a comparative analysis was conducted by measuring its expression levels in twenty pairs of glioma tissues and their corresponding adjacent normal brain tissues using real-time qRT-PCR. According to Fig. 1a,b, miR-410 expression levels were significantly underexpressed in glioma samples when contrasted to adjacent normal brain tissues (*P* < 0.001). Importantly, the level of miR-410 expression was detected to be comparatively reduced in high-grade glioma tissue samples in contrast to low-grade glioma tissue samples (Fig. 1c; *P* < 0.001). Along with glioma tissues, the endogenous expression of miR-410 was observed in a range of glioma cell lines (U251, U87, U373, SHG-44) and a primary human fetal glial cell line HEB. MiR-410 was found markedly decreased in U251, U87, U373, SHG-44 and T98G glioma cells (Fig. 1d; *P* < 0.05). Collectively, the findings indicate that miR-410 exhibits a frequent down-regulation pattern in glioma tissues and cell lines. Since the expression of miR-410 was lower in U87 and SHG-44 cells, the selection of these two cell lines was made for the purpose of conducting subsequent experiments.

### MiR-410 is epigenetically regulated by DNA methylation in glioma

**Figure 2:**
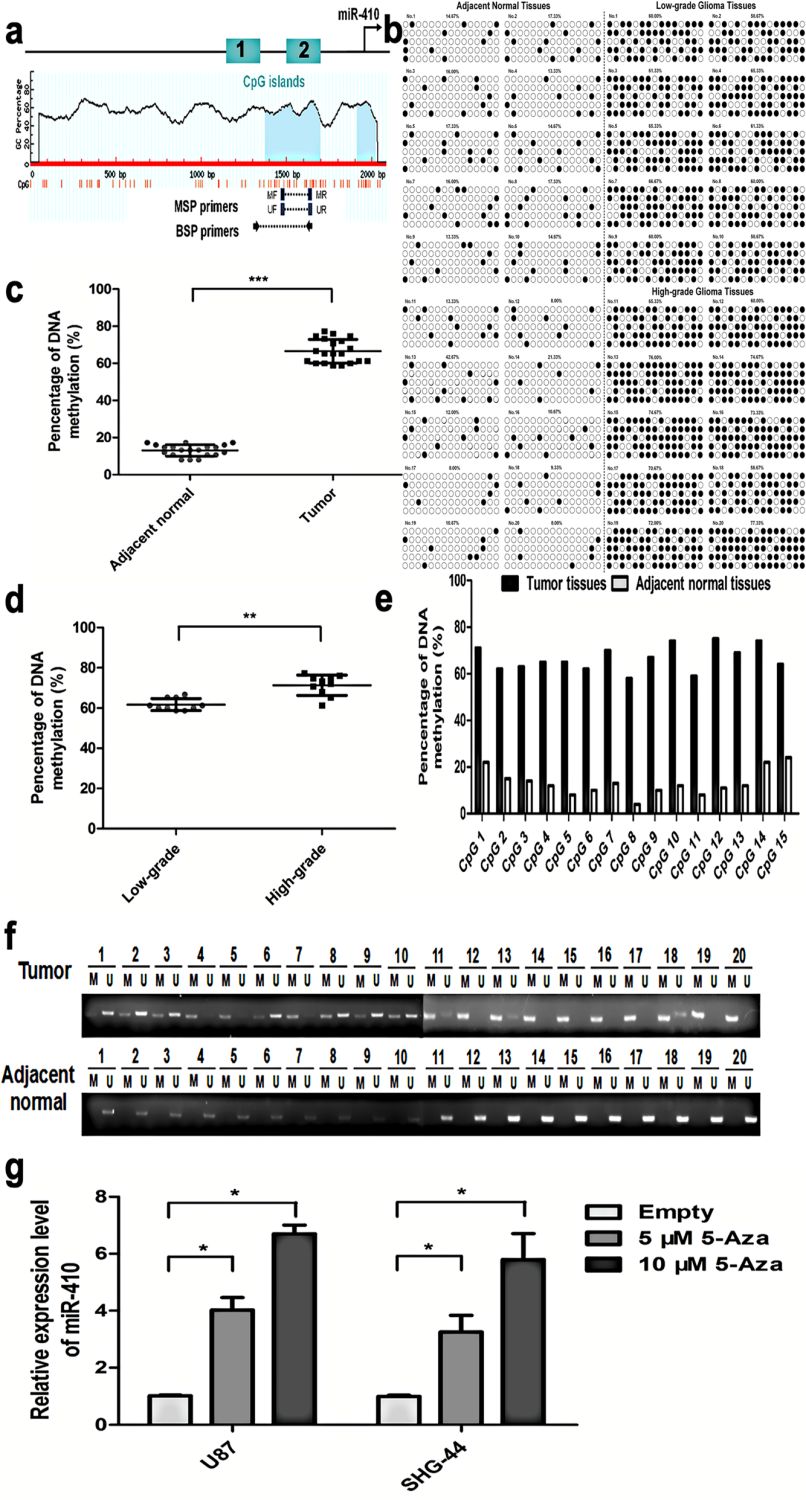
The expression of MiR-410 in glioma is subject to epigenetic regulation through DNA methylation. (**a**) A schematic illustration of CpG islands in the upstream region of miR-410 gene and the primers for MSP or BSP. (**b**) Bisulfite-sequencing PCR identified the methylation status among 20 pairs of glioma tissues (Tumor) and their corresponding adjacent normal tissues (Adjacent normal). Columns represent CpG sites 1–15, and horizontal lines indicate clones 1–5. Black dots symbolize methylated CpG sites, and white dots symbolize unmethylated CpG sites. (**c**) Comparison of methylation levels of miR-410 in glioma and adjacent normal tissues. (**d**) Comparison of methylation levels of miR-410 in different grade tumor tissues (Low-grade or High-grade tumor). (**e**) Comparison of methylation levels of 15 CpG sites in the promoter region of miR-410 in glioma and adjacent normal tissues. (**f**) MSP findings of miR-410 methylation in the 20 cases glioma tissues (10 cases Low-grade tumors or 10 cases High-grade tumors) and their corresponding adjacent normal tissues (Adjacent normal) in pair. M = 172 bp PCR products amplified by MSP specific for methylated DNA, U = 176 bp PCR products amplified by MSP specific for unmethylated DNA. (**g**) Effect of 5-Aza-dC treatment on miR-410 expression in U87 and SHG-44 cells tested by qRT-PCR.

Given that a significant number of miRNAs are subject to regulation by epigenetic modifications, particularly DNA methylation, we proceeded to investigate the regulatory mechanism underlying the expression of miR-410, focusing on its promoter methylation. Two CpG islands were identified within the genomic region upstream of the miR-410 gene (Fig. 2a). In order to explore the epigenetic regulation of miR-410 in glioma, genomic DNA extracted from glioma tissues and corresponding adjacent normal brain tissues was subjected to bisulfite treatment, followed by PCR amplification. The resulting PCR products from each tissue were then cloned into a sequencing vector and subsequently analyzed through bisulfite sequencing. As shown in Fig. 2b, analysis of sequencing findings demonstrated that the proportion of methylation of the promoter region of the miR-410 gene was 84.27% in high-grade glioma tissues (Stage III ~ IV), 72.49% in low-grade glioma tissues (Stage I ~ II), and 33.93% in adjacent normal brain tissues, separately. The statistical analysis findings suggest a significant increase in the methylation rate of the promoter region of miR-410 in glioma tissues when compared to adjacent normal tissues (Fig. 2c; *P* < 0.001). Importantly, the promoter region methylation frequency of miR-410 exhibits a higher incidence in high-grade glioma tissues compared with low-grade glioma tissues (Fig. 2d; *P* < 0.01). The methylation level of CpG loci of miR-410 exhibited a higher value in glioma tissues, whereas it was observed to be reduced in adjacent normal tissues (Fig. 2e). Moreover, the study conducted Methylation-special-PCR on a total of 20 pairs of glioma tissues and their corresponding adjacent normal tissues. The study revealed that DNA methylation was observed in the upstream regions of miR-410 in a significant number of tumor tissues (20 in total), with 13 of them showing partial methylation and 7 showing complete methylation. In contrast, the adjacent normal brain tissues exhibited no methylation (Fig. 2f). Meanwhile, U87 and SHG-44 cells were subjected to treatment with a dimethyltransferase inhibitor known as 5-aza-2’-deoxycytidine (5-Aza-dC). Subsequently, qRT-PCR analysis was conducted, which revealed that the expression of miR-410v was underexpressed in U87 and SHG-44 cells after the 5-Aza-dC treatment contrasted to untreated empty cells in a dose-dependent manner (Fig. 2g), indicated the upstream CpG islands of the miR-410 gene were demethylated following treatment with 5-Aza-dC. Collectively, these findings indicate that epigenetic factors may have an impact on the regulation of miR-410 expression in glioma.

### MiR-410 suppresses glioma cell proliferation and mediates cell apoptosis in vitro

**Figure 3:**
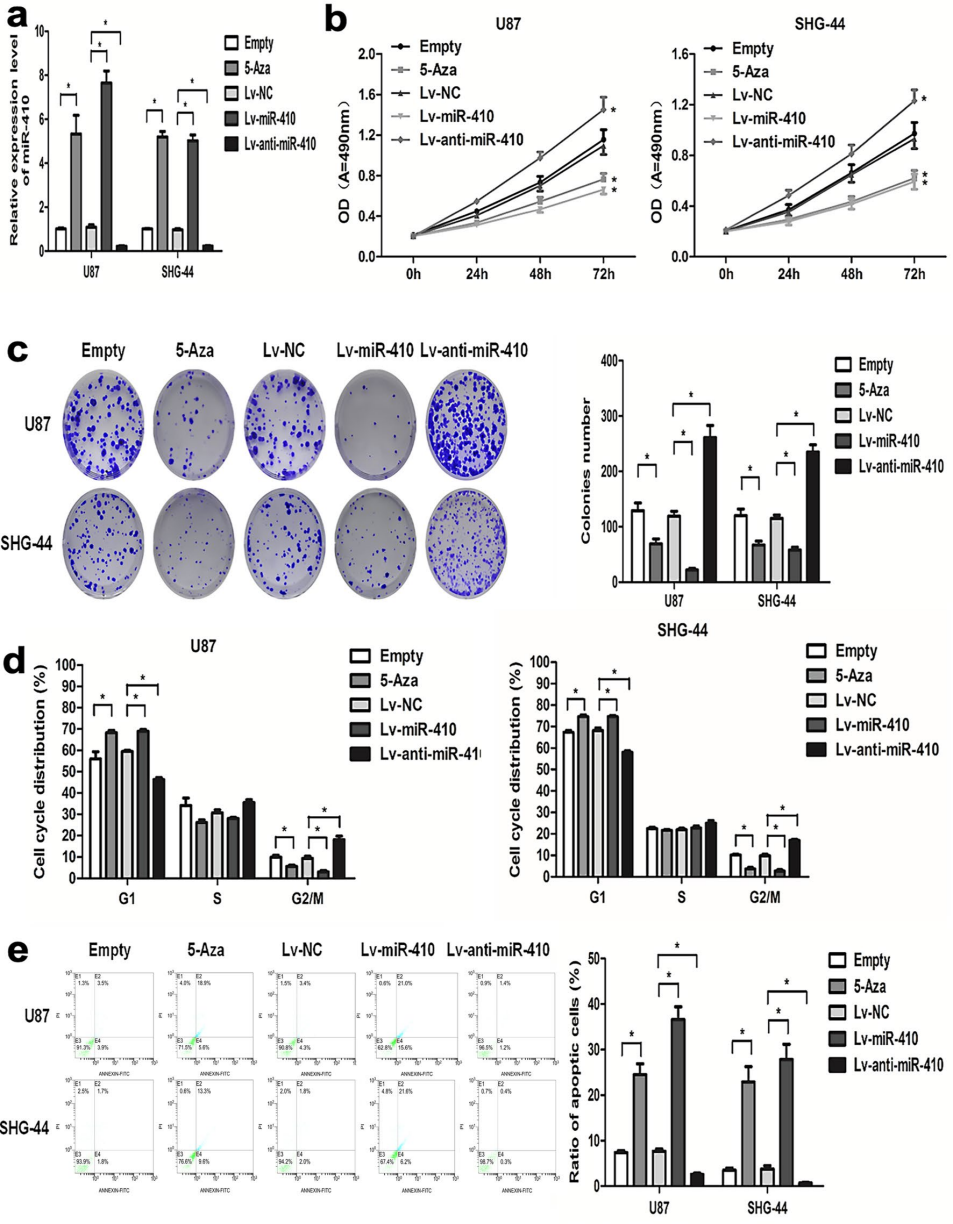
MiR-410 inhibits glioma cell proliferation and mediates cell apoptosis in vitro. (**a**) The relative expression of miR-410 in U87 and SHG-44 glioma cells after lentivirus infection or is determined by qRT-PCR(n = 6). (**b**) MTT assay was employed to investigate cell proliferation of U87 and SHG-44 cells after lentivirus infection or 5-Aza-dC treatment (10 μM) (n = 6). (**c**) The colony formation assay was utilized to examined the formation abilities of U87 and SHG-44 cells after lentivirus infection or 10 μM 5-Aza-dC treatment, respectively(n = 6). (**d, e**) Representative images and quantification of flow cytometry analysis of cell cycle distribution and cell apoptotic ratio of U87 and SHG-44 cells after lentivirus infection or 5-Aza-dC treatment, respectively. Data are shown as mean ± SD of three independent experiments(n = 6). *P < 0.05; **P < 0.001, versus their control, respectively.

To investigate the biological function of miR-410 in glioma cells, we first infected U87 and SHG-44 cells with Lentiviral expressing pre-miR-410 (Lv-miR-410) or miR-410 antisense (Lv-anti-miR-410) to return or suppress the expression of miR-410. qRT-PCR was performed to validate the restoration or inhibition of miR-410 in the stably infected cells (Fig. 3a). Subsequently, an investigation was conducted to examine the impact of miR-410 on cell growth and apoptosis separately. The findings of the MTT assay and the colony formation assay suggest that the upregulation of miR-410 can significantly suppress cellular proliferation and colony formation capacity in U87 and SHG-44 cells, as compared to the control group (Fig. 3b,c). Furthermore, it was observed that the restoration of miR-410 resulted in a significant elevation in the proportion of G1 (not significantly) and G2 phase cells in U87 and SHG-44 cell lines (Fig. 3d; *P* < 0.05) and mediated cell apoptosis (Fig. 3e), as identified by FCM analysis. Conversely, upon silencing of endogenous miR-410, there was a significant elevation in cell progression and colony formation ability, while cell cycle arrest and apoptosis were eliminated (Fig. 3b–d; *P* < 0.05). The findings of the functional analysis indicate that the restoration of miR-410 due to demethylation resulted in the inhibition of cell progression and colony formation ability, stimulation of cell cycle G1 arrest, and promotion of cell apoptosis in U87 and SHG-44 cells (Fig. 3b–d; *P* < 0.05). This investigation outcome indicates that miR-410 could potentially act as a tumor suppressor in vitro by inhibiting cell proliferation and mediating apoptotic processes in glioma cells.

### MiR-410 suppresses glioma cell proliferation and mediates cell apoptosis in vivo

**Figure 4:**
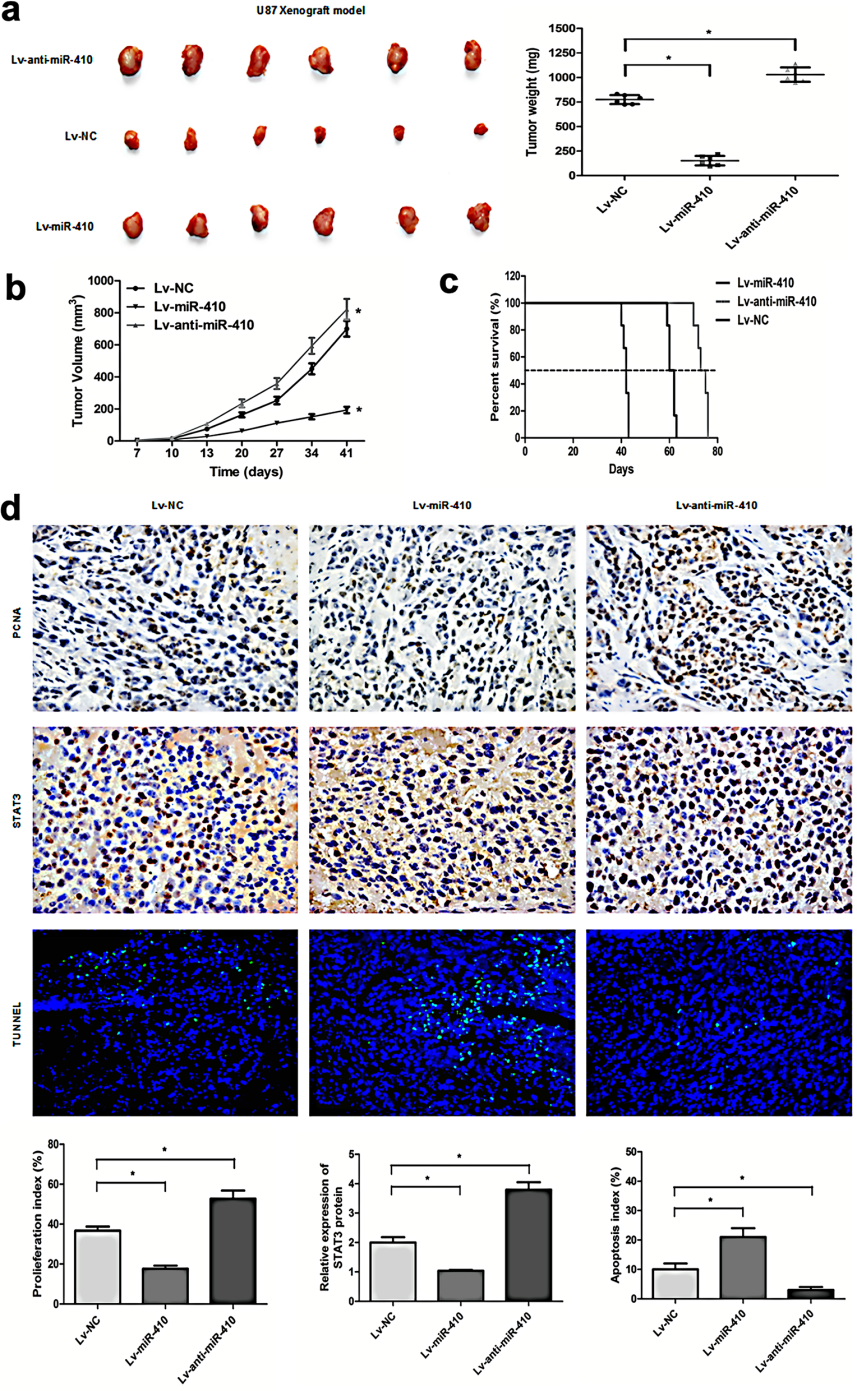
MiR-410 blocks glioma cell growth and mediates cell apoptosis in vivo. (**a**) Photograph of three group tissues and tumor weights dissected from nude mice with the tumor formed by the cells from U87 infected with LV-miR-NC, LV-miR-410 or LV-anti-miR-410(n = 6). (**b**) Three groups measured every 5 days by caliper measurement up to 41 days(n = 6). (**c**) Kaplan–Meier (survival) analysis of three groups with LV-miR-NC, LV-miR-410, and LV-anti-miR-410(n = 6). (**d**) Immunohistochemistry of PCNA, STAT3 staining (× 200) and Tunnel assay (× 200) of the tumor formation of U87 infected with LV-miR-NC, LV-miR-410 or LV-anti-miR-410. *P < 0.05 compared with LV-miR-NC group.

To determine the function of miR-410 in glioma growth in vivo, a xenograft tumor model was developed by directly injecting U87 stable cells infected by Lv-miR-410 or Lv-anti-miR-410 or Lv-NC into the left flanks of nude mice subcutaneously. The previous data revealed that restoration of miR-410 suppressed the proliferation of glioma xenografts and extended the glioma-bearing mice survival time (Fig. 4a–c; *P* < 0.05). The experimental group with overexpression of miR-410 (Lv-miR-410) exhibited a significant decrease in both tumor volume and weight as contrasted with the control group (Lv-miR-NC). In contrast, miR-410 suppression promoted the growth of glioma xenografts in Lv-anti-miR-410 group mice and was negatively associated with a better survival rate (Fig. 4a–c; *P* < 0.05). Immunohistochemistry analysis showed marked decreases of PCNA protein in the LV-miR-410 group but an increase of PCNA protein in the Lv-anti-miR-410 group (Fig. 4d; *P* < 0.05). Furthermore, the tumors of the Lv-miR-410 group exhibited elevated levels of apoptosis as determined by a TUNEL assay. Conversely, the Lv-anti-miR-410 group displayed reduced levels of apoptosis in comparison to the Lv-NC control group (Fig. 4d; *P* < 0.05). Taken together, the current investigation outcomes show that miR-410 impacts glioma cell proliferation and mediates cell apoptosis in vivo.

### MiR-410 directly targets STAT3 and inhibits its expression in glioma cells

**Figure 5:**
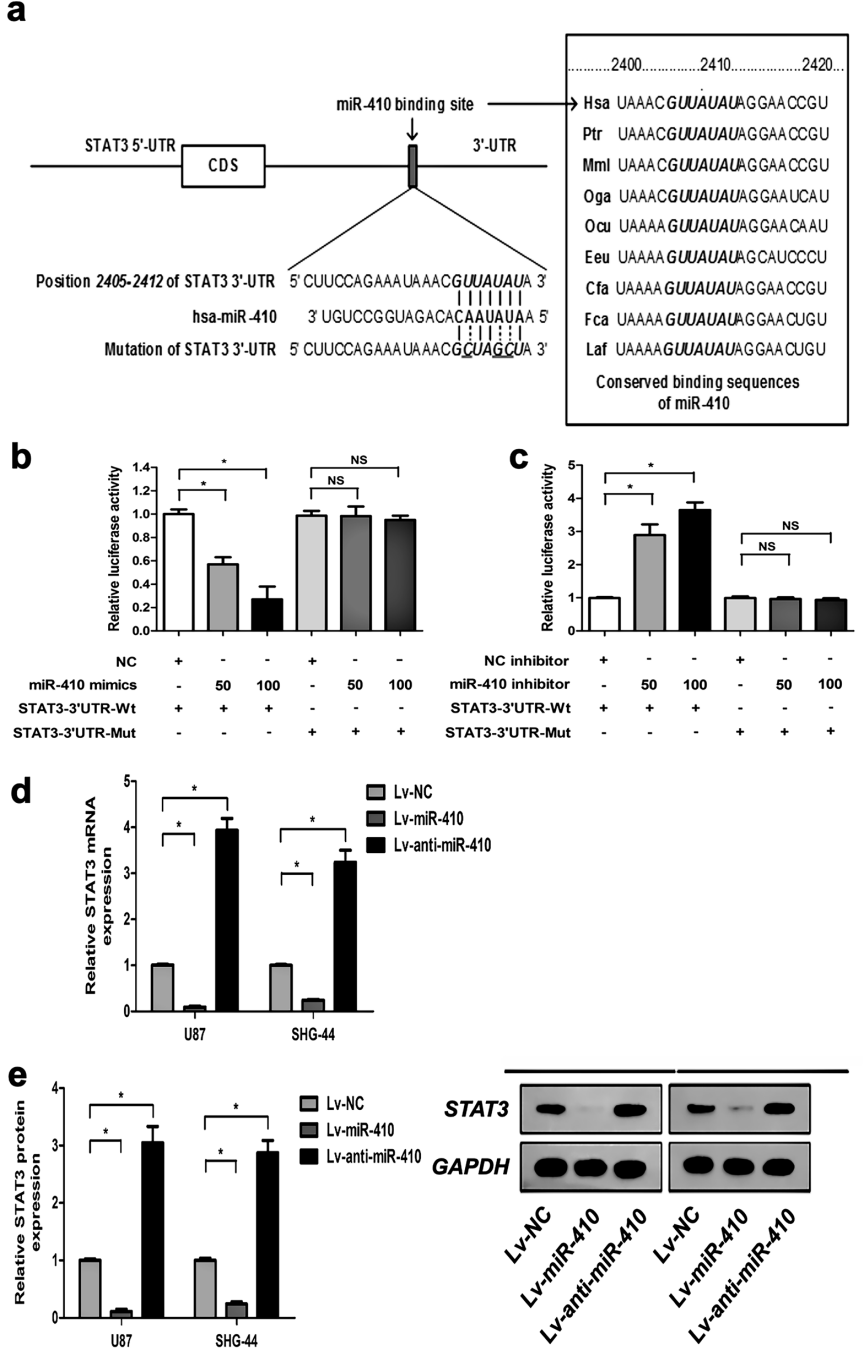
MiR-410 directly targets STAT3 and suppresses its expression in glioma cells. (**a**) Puative binding sequences of miR-410 in the 3’UTR of STAT3. Mutation was produced in the seed region (bold bases) of the 3'UTR of STAT3. (**b**, **c**) The impact of miR-410 (or miR-410 inhibitor) on reporters of STAT3-3’UTR-Wt and STAT3-3’UTR-Mut in U87 and SHG-44 cells by luciferase gene assays. *P < 0.05, contrasted to NC or NC inhibitor. (**d**) RT-PCR showing the levels of STAT3 mRNA in U87 and SHG-44 cells after lentivirus infection or 5-Aza-dC treatment, respectively. GAPDH was utilized as loading control. (**e**) Western blot assay in U87 and SHG-44 cells infected with Lv-NC, Lv-miR-410, and Lv-anti-miR-410(n = 6); GAPDH was utiized as loading control. Data are expressed as mean ± SD of three independent experiments.

In order to explicate the molecular mechanism underlying the inhibitory impact of miR-410 on glioma cells, we utilized three computational algorithms to anticipate potential targets of miR-410 (TargetSan, RNAhybrid, and DIANA-MicroT v3.0). Subsequently, the attention was directed towards STAT3, given its implication in the development of numerous forms of cancers in humans. The current investigation depicts the anticipated associations between miR-410 and the target sites in the 3’-UTR of STAT3 are presented in Fig. 5a. The seed region exhibited complete complementarity (the core sequences that comprise the first 2–8 bases of the mature miRNA), and the miR-410 binding sequences in the STAT3-3’-UTR exhibited a high degree of conservation across various species (Fig. 5a). In contrast, the STAT3 expression was elevated in the Lv-anti-miR-410 glioma cell group contrasted to those in Lv-NC glioma cells as control. In order to conduct a more comprehensive examination of the connection between miR-410 and STAT3 in glioma cells, a luciferase activity assay was carried out (Fig. 5b,c). Consistent with expectations, the data revealed that miR-410 exhibited significant dose-dependent inhibition of firefly luciferase activities of STAT3-Wt. However, in U87 and SHG-44 cells with mutant target sites, miR-410 was found to be ineffective (Fig. 5b). Furthermore, the suppression of endogenous miR-410 through the use of a miR-410 inhibitor led to a notable rise in firefly luciferase activities of STAT3-Wt in U87 and SHG-44 cells. However, this effect was not observed in the mutant, thus providing additional evidence that miR-410 directly targets STAT3 (Fig. 5c).

The western blot and RT-PCR confirmed that the expression of STAT3 was significantly reduced in glioma cells stably transfected with Lv-NC, Lv-miR-410, and Lv-anti-miR-410, respectively. The results demonstrated that overexpression of miR410 in both U87 and SHG-44 cell lines can effectively and significantly reduced the expression of STAT3 mRNA and protein. Conversely, the use of anti-miR410 significantly increased the expression levels of STAT3 mRNA and protein(Fig. 5d,e). The results of this investigation indicate that miR-410 exerts direct regulatory control over STAT3 and suppresses its expression within glioma cells.

### STAT3 is included in the modulation of cell growth, cell cycle distribution, and apoptosis of glioma cells by miR-410

**Figure 6:**
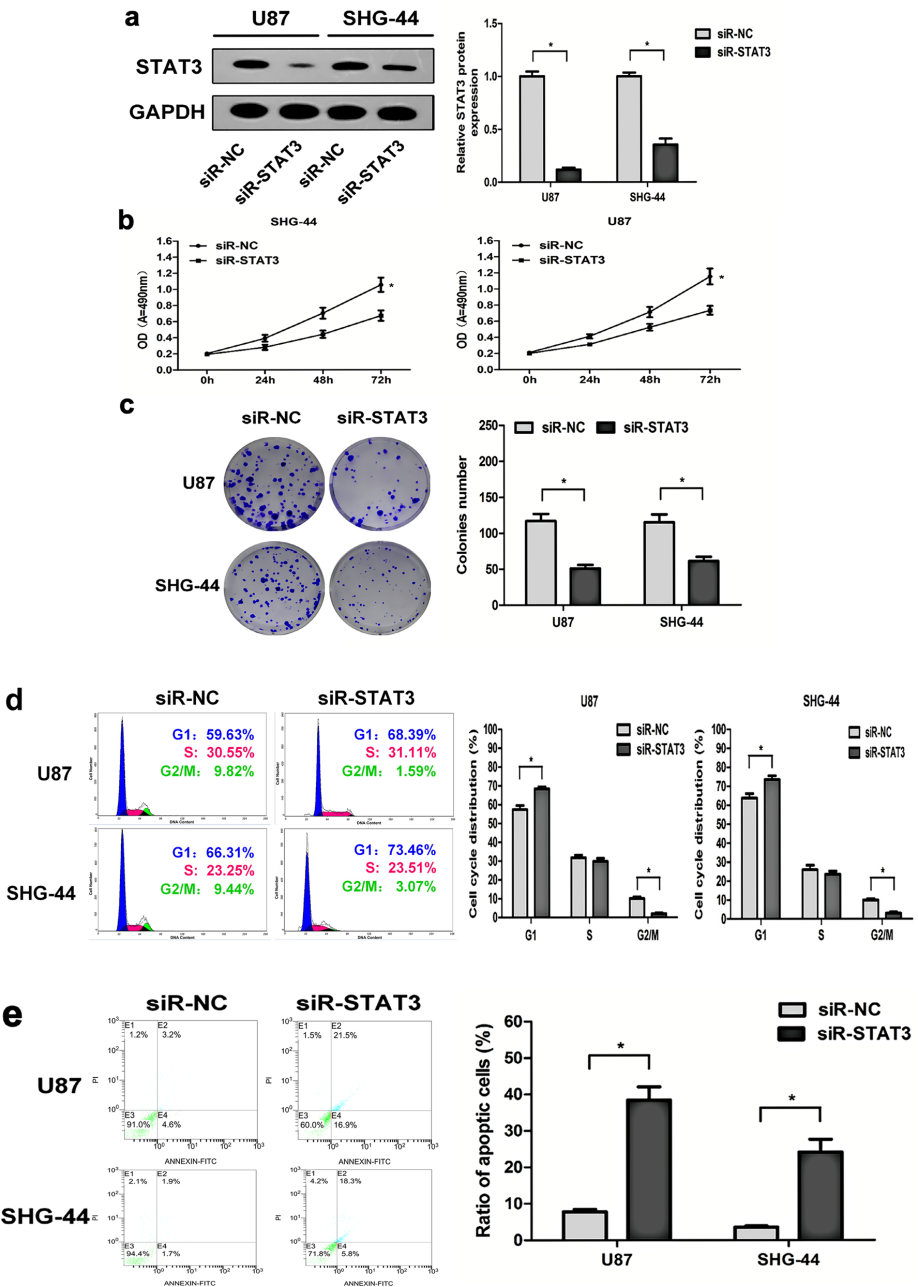
Knock-down of STAT3 suppresses cell growth, prompts cell apoptosis of glioma cells in vitro. (**a**) U87 and SHG-44 cells underwent infection with STAT3-specific small interfering RNAs (siR-STAT3) and negative control (siR-NC), and STAT3 expression levels were investigated by Western blot(n = 6). (**b, c**) The impact of STAT3 knock-down on the cell proliferation and colony formation abilities was evaluated by MTT assay and colony formation assay, respectively(n = 6). (**d, e**) The impacts of STAT3 knock-down on the cell cycle and cell apoptosis were evaluated by flow cytometry analysis, respectively(n = 6). *P < 0.05 versus the control, data are shown as mean ± SD of three independent experiments.

To conduct a more in-depth investigation into the potential correlation between the modulation of STAT3 and the functional roles it plays in glioma cells. The STAT3 gene was suppressed in U87 and SHG-44 cells through the use of STAT3-specific small interfering RNAs (siR-STAT3), as verified by qRT-PCR (Fig. 6a). Subsequently, the MTT assay was conducted (Fig. 6b), colony formation assay (Fig. 6c), cell cycle and cell apoptosis analysis (Fig. 6d,e) revealed that knock-down of STAT3 significantly inhibited cell proliferation and the capability of colony formation stimulated the cell cycle G1 arrest and promoted the cell apoptosis in U87 and SHG-44 cells, respectively. The findings indicate that the reduction of STAT3 expression exhibited a partial phenocopy to the impacts of miR-410 upregulation in glioma cells. Collectively, the outcomes indicate that miR-410 exerts a suppressive impact on the growth of glioma, in part, by directing its focus towards STAT3.

### Upregulation of STAT3 is negatively associated with miR-410 expression in clinic glioma tissues

**Figure 7:**
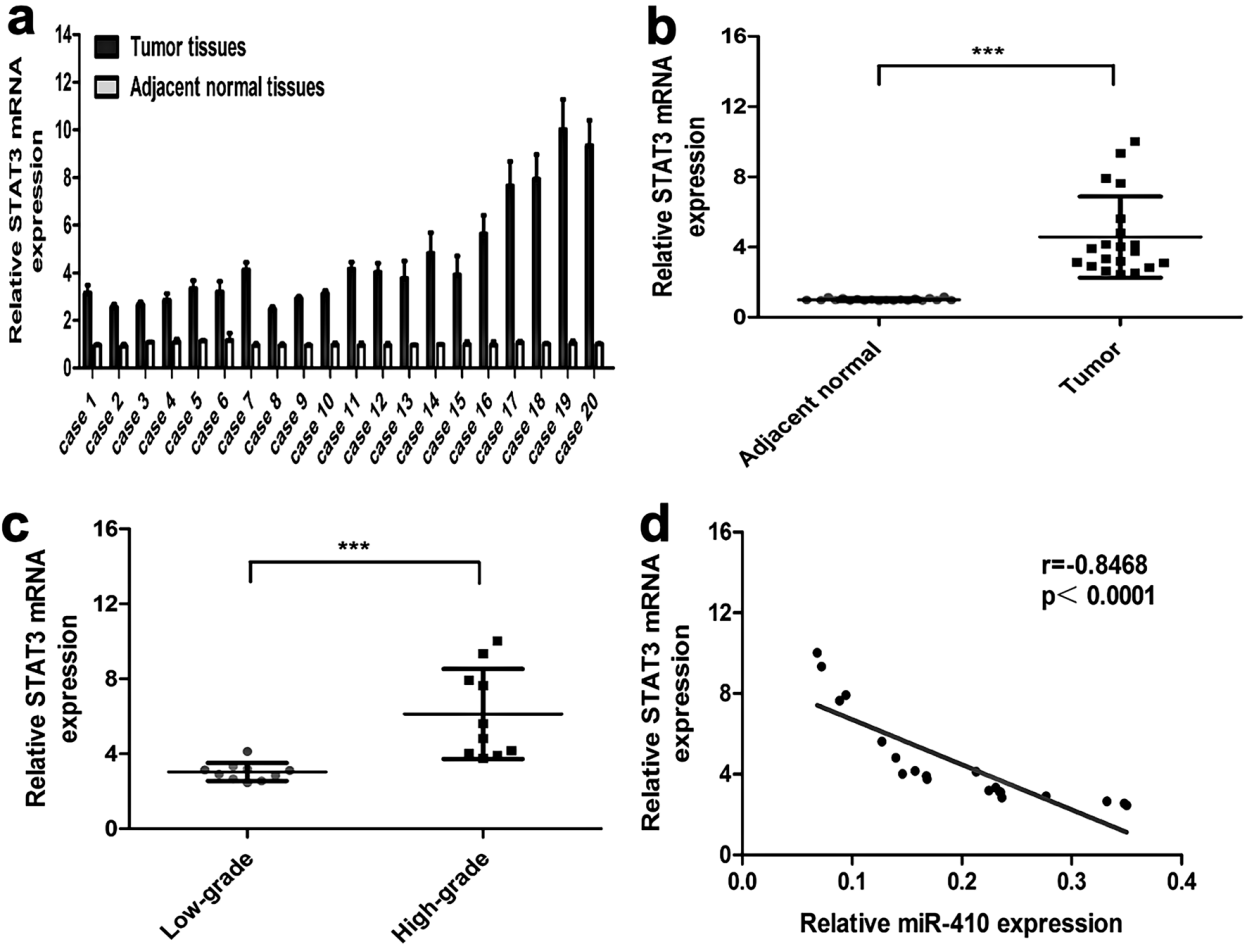
Expression of STAT3 is increased, negatively associated with miR-410 in clinic glioma samples. (**a**) qRT-PCR was utilized to detect the mRNA expression levels of STAT3 in 20 pairs of glioma tissues and their corresponding adjacent non-plastic brain tissues. (***P < 0.001, n = 6). (**b**) Quantification of the relative levels of mRNA expression of STAT3 in 20 pairs of glioma tumor tissues and their corresponding adjacent tissues based on qRT-PCR. (**c**) qRT-PCR analysis of STAT3 in glioma tissues with different stages. All data were normalized to GAPDH. (***P < 0.001, n = 6). (**d**) Spearman's correlation Analysis among miR-410 and STAT3 mRNA expression in 20 pairs of glioma tumor tissues (*P* < 0.001, r = -0.8468).

qRT-PCR was employed in the present study to evaluate STAT3 expression levels in twenty sets of clinical glioma tissues and their corresponding normal brain tissues. In glioma tissues, there was an observed increase in STAT3 mRNA expression contrasted with their corresponding adjacent normal counterparts (Fig. 7a,b; *P* < 0.001). Moreover, the findings indicate a statistically significant upregulation of STAT3 mRNA expression within high-grade gliomas compared to low-grade gliomas (Fig. 7c; *P* < 0.05). Consistent with miR-410 suppression of STAT3 in cultivated glioma cells, a significant negative correlation was detected between the mRNA levels of STAT3 and the levels of miR-410 in glioma samples (Fig. 7d; *P* < 0.001, *r* = -0.8865). Collectively, the data strongly indicate significant pathological implications of miR-410 and STAT3 in glioma.

## Discussion

Dysregulated expression of miR-410 has been observed in diverse types of cancer. Similar to other miRNAs, miR-410 acts as a bi-functional gene either as an oncogene or tumor suppressor, which largely depends on the cancer type and genetic context^11^. For example, miR-410 exhibited upregulation in liver and colorectal tumors, thereby promoting the progression and invasion of cancerous cells via directly/indirectly regulating tumor-suppressor gene FHL1. Yuan et al. also revealed that miR-410 was observed to exhibit oncogenic properties in non-small cell lung cancer via targeting PI3K/mTOR pathway^12^. In addition, underexpression of miR-410 expression is also detected in breast cancer, pancreatic cancer, osteosarcoma, and pituitary gonadotroph tumors, supporting the tumor suppressive function of miR-410 in these cancers^13^. Throughout the present investigation, we discovered that miR-410 was significantly decreased in clinic glioma tissues and cultivated glioma cells contrasted to those in the corresponding adjacent normal brain tissues and HEB cell lines, respectively. In particular, the current investigation revealed that the levels of miR-410 were comparatively decreased in high-grade glioma tissues contrasted to low-grade glioma tissues, which indicated that miR-410 has the ability to act as a novel biomarker for determining the pathological grade of glioma. Furthermore, our study revealed that the upregulation of miR-410 in glioma cell lines significantly inhibited cell proliferation and cell colony formation, arrested cell cycle at G1 phase, and prompted cell apoptosis by gain of functional experiments in vitro. Our results were in line with the report that miR-410 could modulate MET to affect the glioma cells growth and invasion. Moreover, the current investigation revealed that miR-410 also has anti-tumor activity in the mouse glioma xenograft model. Overexpression miR-410 significantly inhibited cell growth of glioma tumors in mice. Decreased PCNA protein level, which is a marker for cell proliferation. A TUNEL assay demonstrated that miR-410 stimulated apoptosis in vivo. Taken together, these findings indicate that miR-410 performs a crucial function in glioma development and proliferation.

The epigenetic suppression of miRNAs through DNA methylation has been established in various cancer types, with aberrant DNA methylation being a primary contributor to miRNA dysfunction in cancer^14^. Nevertheless, there is a restricted number of studies that have investigated the DNA methylation of miRNAs in glioma, and DNA methylation affecting miR-410 in glioma has remained poorly studied^15^. We then analyzed 1 kb upstream of the transcription start site of miR-410 and found two high-density CpG regions referred to as CpG Islands (CGIs). Using bisulfite sequencing, we explored that therein 15 CpG sites in the CGIs of miR-410 gene upstream had a higher frequency of methylation within glioma tissues contrasted with their matched adjacent normal tissues, which were consistent with the results of Methylation-specific PCR in glioma tissues and their matched adjacent normal tissues. Moreover, the frequency of methylation in high-grade glioma tissues was greater than that in low-grade glioma tissues, which indicated that methylation levels might perform a crucial role as a new diagnostic and prognostic marker in glioma. In addition, we analyzed methylation and expression of miR-410 in the presence of demethylation drugs (5-Aza-dC) and found that methylation of miR-410 showed a dramatic reduction, whereas miR-410 expression exhibited a significant increase after treatment with 5-Aza-dC in two of the glioma cell lines. Previous studies have shown that 5-aza-dC does not increase miR-410 expression in human leukemia K562 cells. We suggested that the epigenetic regulation of miR-410 may happen in a tissue-specific and/or cancer-type-specific manner in cancer-distinct forms. Throughout the current investigation, our data strongly suggested that miR-410 expression was relevant to epigenetic regulation, including promoter methylation, at least in glioma cancer cells tested in this investigation. Additionally, our research showed that 5-Aza-dC treatment significantly induced inhibition of cell progression and cell colony formation, arrest of the cell cycle, and promotion of cell apoptosis in glioma cells. Different from genetic alterations, DNA methylations are reversible. Hyper-methylated suppressor genes can be re-activated by demethylating agents, suggesting epigenetic change as a target of tumor therapy. DNA demethylating drugs, which have been approved for therapeutic usage, are clinically effective against leukemia and myelodysplastic syndrome^16^. Nevertheless, the clinical effectiveness of demethylating drugs in treating solid tumors has yet to be established. The study demonstrated a significant potential advantage of epigenetic therapy through the modulation of miRNA expression in individuals diagnosed with glioma ^17^.

Currently, there exists a limited number of reported targets of miR-410. Acuna et al. discovered that miR-410 negatively regulates pRb/E2F pathway by directly targeting the oncogene CDK1 in cancer cells^18^. Another study found that miR-410 inhibits cell growth and invasion by directly targeting vascular endothelial growth factor (VEGF) in osteosarcoma^19^. The precise molecular mechanism by which miR-410 affects glioma remains to be accomplished. The current investigation purpose is to determine the putative target genes of miR-410 in glioma through bioinformatics analysis. Our findings revealed a Signal transducer and activator of transcription (STAT3) as a direct target gene of miR-410, as confirmed by luciferase reporter assay. Furthermore, the findings of western blot and immunochemistry assays demonstrated that up-regulation of miR-410 in glioma cells can inhibit STAT3 expression in vitro and in vivo. In contrast, the STAT3 expression was elevated when endogenous miR-410 in glioma cells was reduced. Signal transducer and activator of transcription (STAT) proteins are a family of transcription factors whose activity is related to a wide variety of biological processes, such as cell development, stem cell differentiation, inflammation, and tumor progression^20^. The STAT3 protein, a significant member of the STAT group, is the favored downstream target of phosphorylated JAK2 and is persistently stimulated in different human malignancies, exhibiting oncogenic properties. Activated STAT3 contributes to tumorigenesis by promoting cell proliferation and impairing host tumor immunity. Wu et al. have sugested that the activation of STAT3 can trigger the overexpression of cyclin D1, leading to the sustained proliferation of malignant cells in cutaneous squamous cell carcinoma tissues^21^. Another investigation has demonstrated that silence of the expression of Stat3 suppresses the proliferation of gastric tumors, and induces cell apoptosis and cell cycle shift induction both in vivo and in vitro significantly by regulating downstream protein Cyclin-D1, Survivin, and Bcl-2. The level of Bcl-XL, another gene in the Bcl-2 gene family, also relied on the sustained activation of STAT3^22^. Research on gastric and pancreatic cancer has produced similar results, confirming these outcomes^23^. Suppressing the signal transduction of STAT3 causes a decrease in the expression of survivin, which in turn makes it possible to stimulate apoptosis in cancer cells^24^. In our investigation, we found that the knock-down of STAT3 suppressed the growth and mediated apoptosis, which effects similar to that in glioma cells when miR-410 overexpression. Furthermore, we confirmed that the upregulation of STAT3 levels is negatively associated with decreased miR-410 expression in glioma tissues. The present study outcomes revealed that miR-410 exerted a suppressive impact on glioma proliferation and apoptosis partially by targeting STAT3. However, our study still has some limitations, such as our oversight of the dual functionality of miRNA methylation in activation or inhibition, as well as issues related to sample size and grouping. On the other hand, we will expand our set of biomarkers for analysis. Within the gene/protein interaction network inside tumor cells, besides the regulation of miR-410/STAT3, there exists a range of proteins, such as MET, the receptor for hepatocyte growth factor receptor (HGF) , which are critical in modulating the biological functions of tumor cells^25^. Additionally, phosphorylated STAT3 (p-STAT3) exhibits a more pronounced regulatory role compared to the unphosphorylated form. Therefore, in our forthcoming research, we plan to enhance our study by increasing sample size and refining our grouping strategy. The expression of phosphorylated forms of several key proteins, in addition to p-STAT3, will be investigated to provide a more comprehensive view of our research in the future. We appreciate your valuable input, which encourages us to further improve our study.

## Conclusion

Finally, it has been determined that the miR-410 expression is commonly suppressed in glioma, and this suppression is inversely associated with the degree of miR-410 methylation. The direct targeting of STAT3 by miR-410 has been demonstrated, and the overexpression of STAT3 in glioma tissues has been linked to the epigenetic suppression of miR-410. By targeting oncogene STAT3, elevated miR-410 activity demonstrated efficacy in suppressing the progression of glioma cancer cells via the stimulation of G1 arrest and in stimulating apoptosis of glioma cancer cells. Our results indicated that epigenetically promoted silencing of miR-410 participates in oncogene STAT3 induction in glioma. Thus, the combined epigenetic and miRNA-based therapies may provide a potential strategy for therapeutic intervention in glioma.

### Supplementary Information

Supplementary Information.

## Data Availability

All data generated or analysed during this study are included in this published article and supplementary information files.
