# Supplementary material for: DNA methylation-mediated repression of microRNA-410 promotes the growth of human glioma cells and triggers cell apoptosis through its interaction with STAT3
Source: Sci Rep. 2024 Jan 18;14:1556. doi: 10.1038/s41598-024-51976-x (PMC10796673; doi:10.1038/s41598-024-51976-x)

1. Table 1 Primer sequence

BSP-Primer-F:	5’-TTGGATTTGGTGATTGGGAA-3'
BSP-Primer-R	5’-ACATTAAAAACAATTACTTTCCAACT-3'
miR-410-MF	5’-CGGTATTAAATTTTATTAGGGAGGTC-3'
miR-410-MR	5'-ACAAACTACAACGAATACGAACGA-3'
miR-410-UF	5’-TTTGGTATTAAATTTTATTAGGGAGGTT-3'
miR-410-UR	5’-CCACAAACTACAACAAATACAAACAA-3'
miR-410-RT	5’-GTCGTATCCAGTGCAGGGTCCGAGGTATTCGCACTGGATACGACACAGGCCA-3’
miR-410-F:	5’-GTCAGCGCAATATAACACAG-3’
U6-RT	5’-GTCGTATCCAGTGCAGGGTCCGAGGTATTCGCACTGGATACGACAAAAATAT-3’
U6-F	5’-GTCAGCGCGTGCTCGCTTCG-3’
β-actin-F	5’-AGGGGCCGGACTCGTCATACT-3’
β-actin-R	5’-GGCGGCACCACCATGTACCCT-3’
STAT3-F	5’-CCCCATACCTGAAGACCAAGT-3’
STAT3-R	5’-TAACTCTCACCCAGTGTCCCA-3’
U6-R	5’-GTGCAGGGTCCGAGGT-3’

2. The designation of primers

The design of primers for molecular biology experiments, such as PCR (Polymerase Chain Reaction) or DNA sequencing, involves several considerations to ensure their specificity and efficiency. Here is a general overview of how primers are typically designed:

a). Identify the Target Sequence: Determine the RNA region to amplify or sequence.
b). Analyze the Target Sequence: Use bioinformatics tools and software to analyze the target sequence. Look for regions that are suitable for primer binding, avoiding repetitive sequences, secondary structures, or regions with high GC or AT content that might cause primer-dimer formation or poor amplification.
c). Select Primer Parameters: Decide on the primer length, typically between 18 to 25 bases, and the optimal melting temperature (Tm) for your primers. The Tm should be calculated to ensure that the primers will anneal specifically to the target sequence.
d). Primer Design Software: Utilize primer design software or online tools that can automatically generate potential primer sequences based on the parameters. These tools often consider factors like Tm, GC content, and primer-dimer formation.
e). Check for Specificity: Verify the specificity of the primers by using BLAST to ensure that they only match the intended target sequence and do not have significant homology with other sequences in the genome or transcriptome.
f). Order and Test: Once primers have been designed, order them from a reputable supplier. Before using them in experiments, perform validation tests, such as standard PCR or qPCR, to ensure they amplify the target sequence efficiently and specifically.

3. Bioinformatics analysis of the relationship between STAT3 and miR-410

miRDB

There are 1118 predicted targets for hsa-miR-410-3p in miRDB.

Target Detail	Target Rank	Target Score	miRNA Name	Gene Symbol	Gene Description
Details	1	99	hsa-miR-410-3p	NUMB	NUMB, endocytic adaptor protein
Details	2	99	hsa-miR-410-3p	NDFIP2	Nedd4 family interacting protein 2
Details	3	99	hsa-miR-410-3p	TRPC1	transient receptor potential cation channel subfamily C member 1
Details	4	99	hsa-miR-410-3p	TENT5A	terminal nucleotidyltransferase 5A
Details	5	99	hsa-miR-410-3p	TBX4	T-box 4
Details	6	99	hsa-miR-410-3p	CREBZF	CREB/ATF bZIP transcription factor
Details	7	99	hsa-miR-410-3p	PRDM11	PR/SET domain 11
Details	8	98	hsa-miR-410-3p	STARD13	StAR related lipid transfer domain containing 13
Details	9	98	hsa-miR-410-3p	ATRX	ATRX, chromatin remodeler
Details	10	98	hsa-miR-410-3p	TMEM108	transmembrane protein 108
Details	11	98	hsa-miR-410-3p	TMEM106B	transmembrane protein 106B
Details	12	98	hsa-miR-410-3p	PDE4B	phosphodiesterase 4B
Details	13	98	hsa-miR-410-3p	CREB5	cAMP responsive element binding protein 5
Details	14	98	hsa-miR-410-3p	ZBTB44	zinc finger and BTB domain containing 44
	⋮			⋮	
Details	713	64	hsa-miR-410-3p	CBX4	chromobox 4
Details	714	64	hsa-miR-410-3p	TTC17	tetratricopeptide repeat domain 17
Details	715	64	hsa-miR-410-3p	PTF1A	pancreas associated transcription factor 1a
Details	716	63	hsa-miR-410-3p	FAM122B	family with sequence similarity 122B
Details	717	63	hsa-miR-410-3p	TENT2	terminal nucleotidyltransferase 2
Details	718	63	hsa-miR-410-3p	MARCH5	membrane associated ring-CH-type finger 5
Details	719	63	hsa-miR-410-3p	PIGK	phosphatidylinositol glycan anchor biosynthesis class K
Details	720	63	hsa-miR-410-3p	TNRC18	trinucleotide repeat containing 18
Details	721	63	hsa-miR-410-3p	MECP2	methyl-CpG binding protein 2
Details	722	63	hsa-miR-410-3p	EPS8	epidermal growth factor receptor pathway substrate 8
Details	723	63	hsa-miR-410-3p	RBL2	RB transcriptional corepressor like 2
Details	724	63	hsa-miR-410-3p	SERPINE2	serpin family E member 2
Details	725	63	hsa-miR-410-3p	STAT3	signal transducer and activator of

miRDB

Gene 6774 is predicted to be targeted by 189 miRNAs in miRDB.

Target Detail	Target Rank	Target Score	miRNA Name	Gene Symbol	Gene Description
Details	1	97	hsa-miR-1299	STAT3	signal transducer and activator of transcription 3
Details	2	95	hsa-miR-374a-3p	STAT3	signal transducer and activator of transcription 3
Details	3	95	hsa-miR-7160-5p	STAT3	signal transducer and activator of transcription 3
Details	4	94	hsa-miR-21-5p	STAT3	signal transducer and activator of transcription 3
Details	5	93	hsa-miR-590-5p	STAT3	signal transducer and activator of transcription 3
Details	6	92	hsa-miR-6825-5p	STAT3	signal transducer and activator of transcription 3
Details	7	92	hsa-miR-4268	STAT3	signal transducer and activator of transcription 3
Details	8	91	hsa-miR-196a-1-3p	STAT3	signal transducer and activator of transcription 3
		⋮	⋮		
Details	90	64	hsa-miR-3157-3p	STAT3	signal transducer and activator of transcription 3
Details	91	64	hsa-miR-3158-5p	STAT3	signal transducer and activator of transcription 3
Details	92	64	hsa-miR-1244	STAT3	signal transducer and activator of transcription 3
Details	93	63	hsa-miR-410-3p	STAT3	signal transducer and activator of transcription 3
Details	94	63	hsa-miR-4441	STAT3	signal transducer and activator of transcription 3
Details	95	63	hsa-miR-29c-3p	STAT3	signal transducer and activator of transcription 3
Details	96	63	hsa-miR-29a-3p	STAT3	signal transducer and activator of transcription 3
Details	97	63	hsa-miR-4700-5p	STAT3	signal transducer and activator of transcription 3

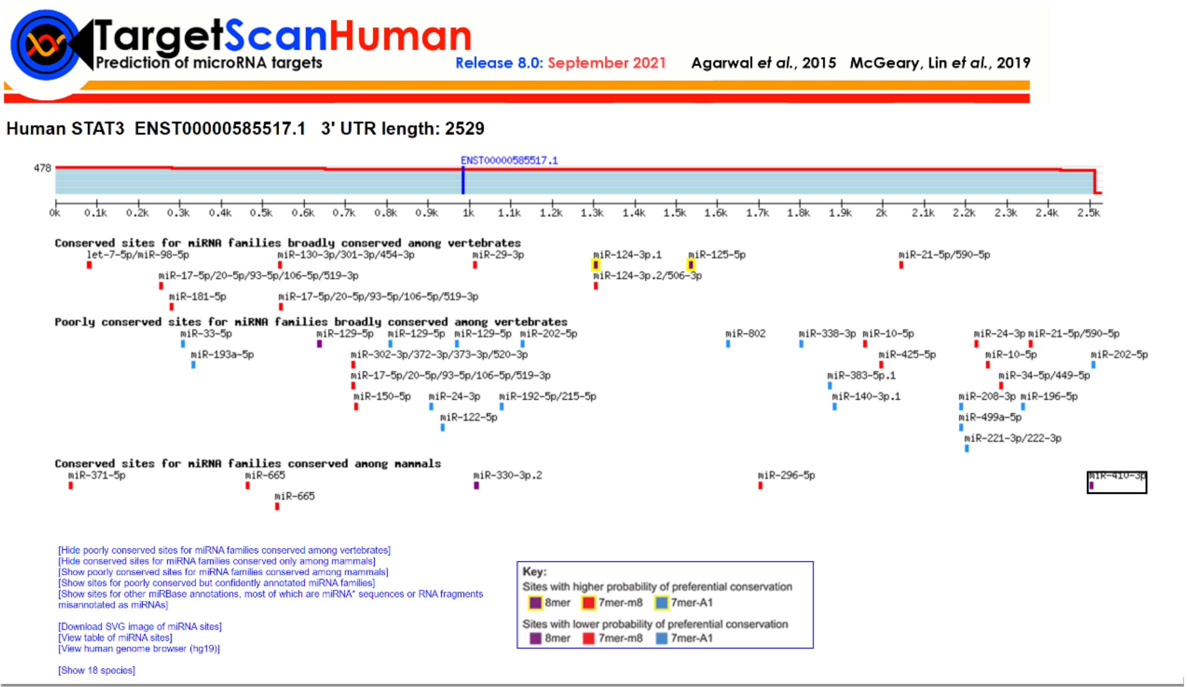

[Download table]

Conserved

	Predicted consequential pairing of target region (top) and miRNA (bottom)	Site type	Context++ score	Context++ score percentile	Weighted context++ score	Conserved branch length	P_{CT}	Predicted relative K_D
Position 2499-2506 of STAT3 3' UTR<br>hsa-miR-410-3p	5' ...CUUCCAGAAAUAAACGUUAUAUA...<br>3' UGUCCGGUAGACACAAUAUAA	8mer	-0.05	91	-0.04	2.900	N/A	-4.021

Context++ score and features that contribute to the context++ score are evaluated as in Agarwal et al., 2015.
Conserved branch lengths and P_{CT} are evaluated as in Friedman et al., 2008, with an expanded 84-species alignment as described in Agarwal et al., 2015.
Predicted relative K_D is evaluated as in McGeary, Lin et al., 2019.

4. Figures

Figure 2f:

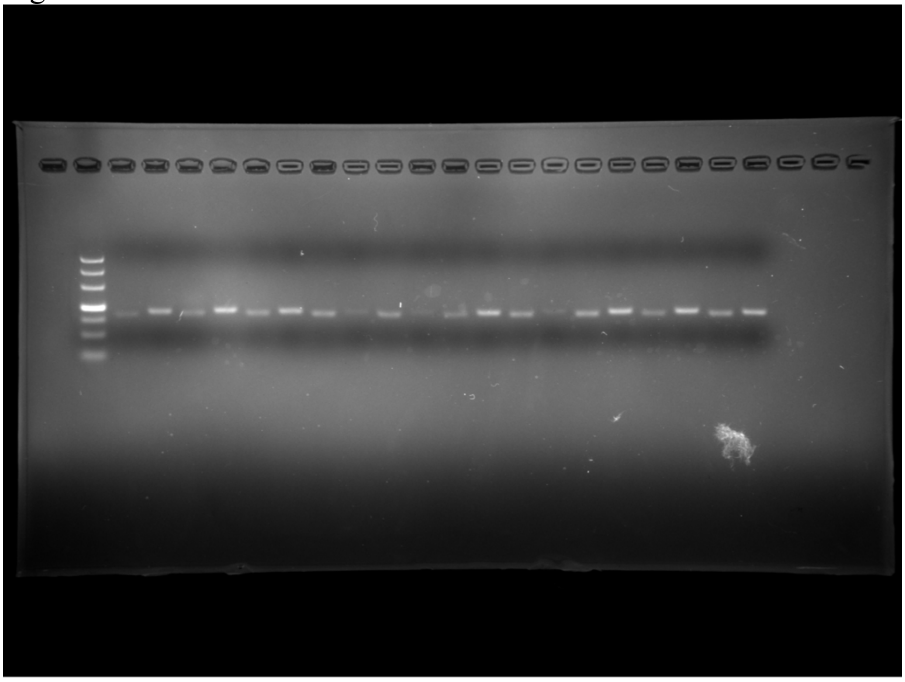

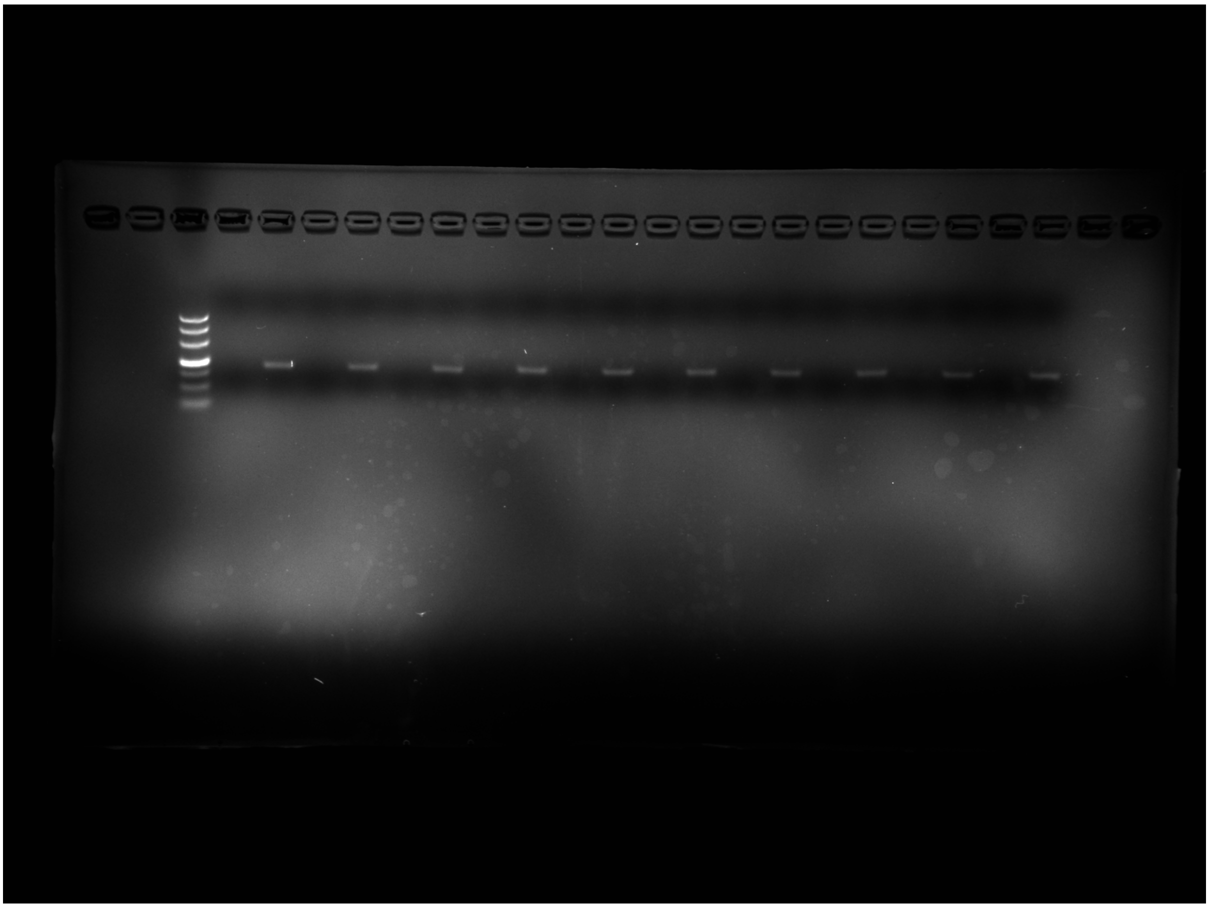

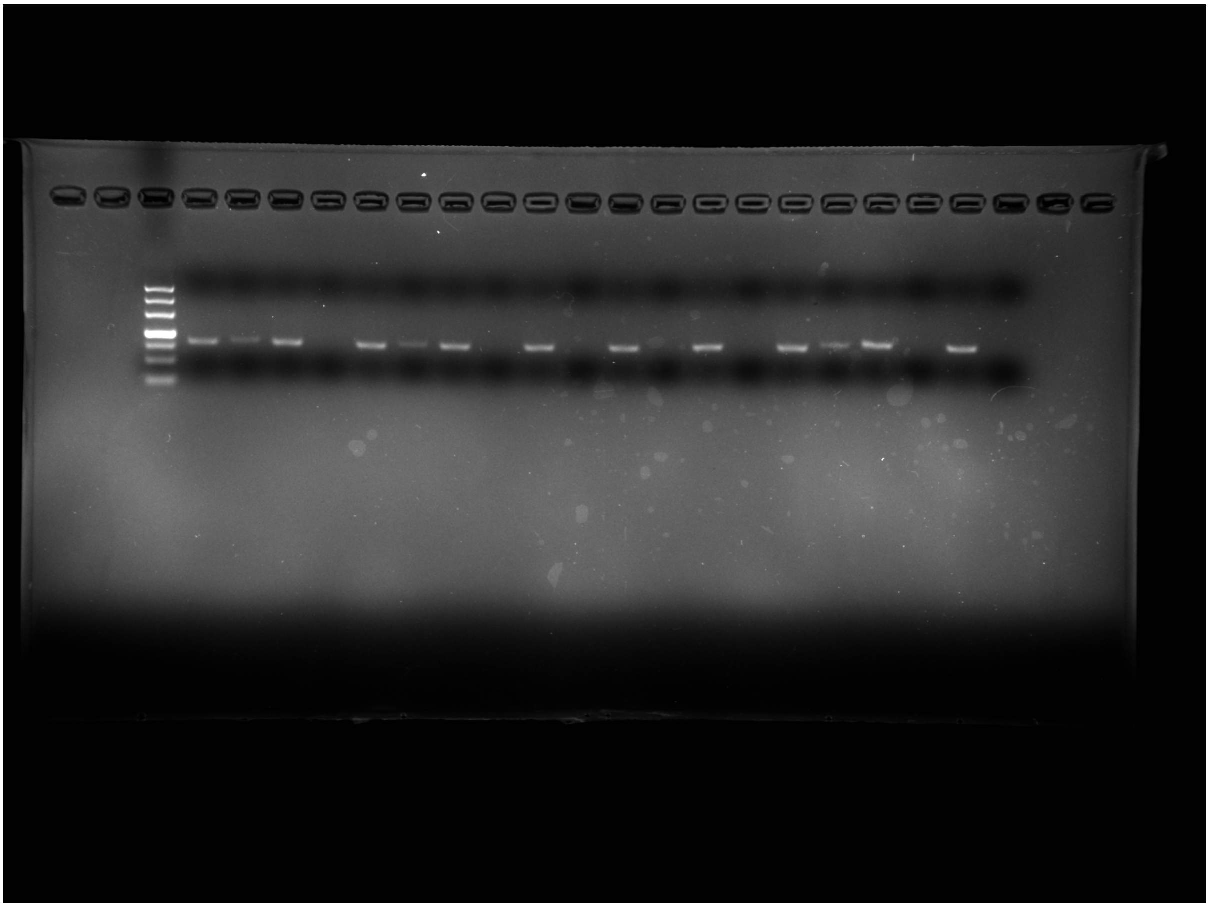

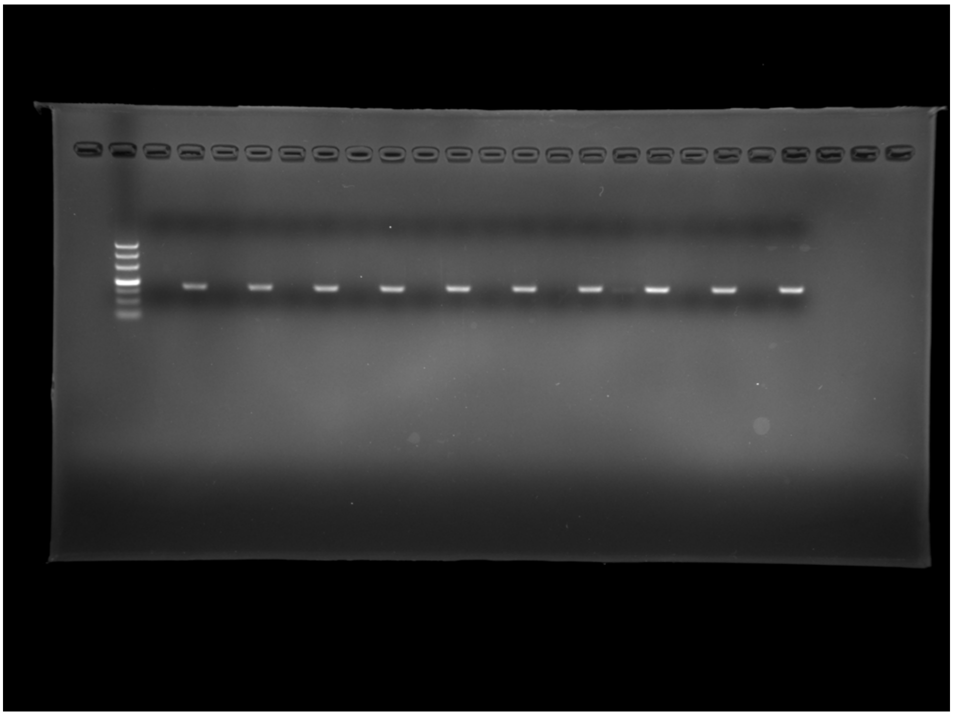

Figure 5e:

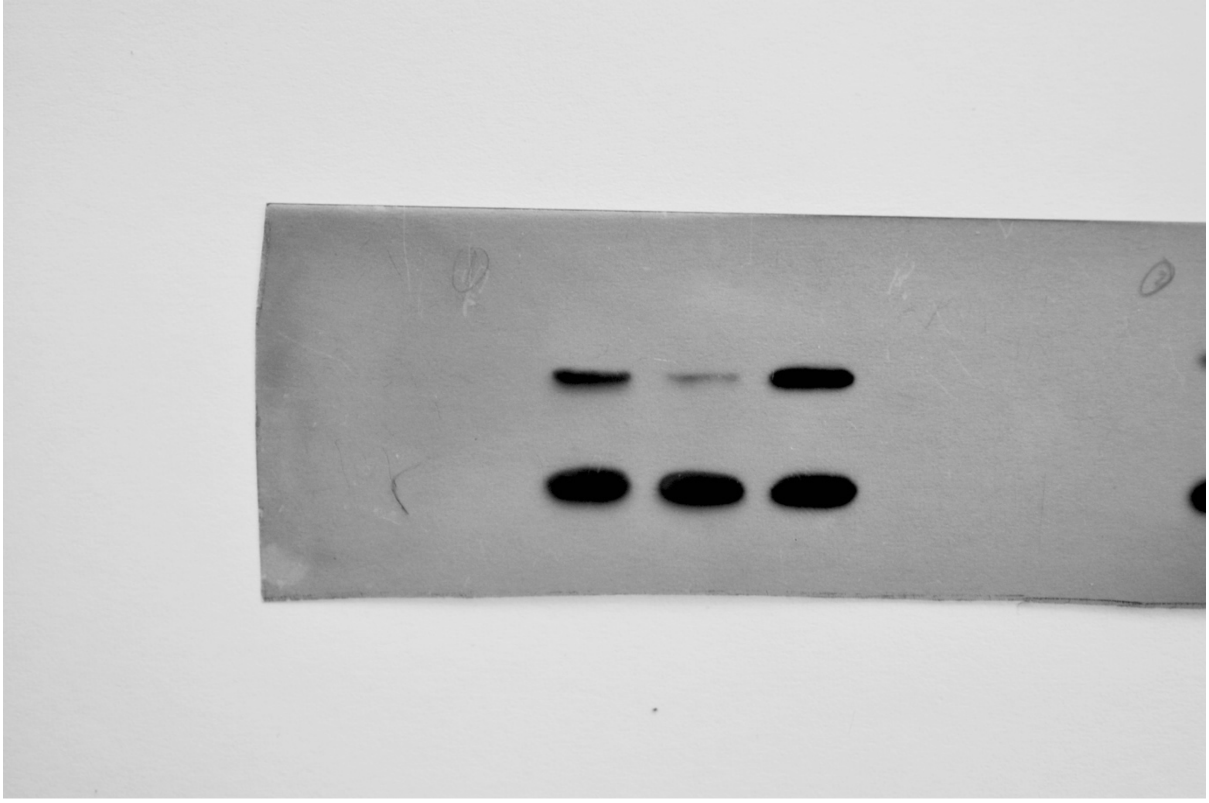

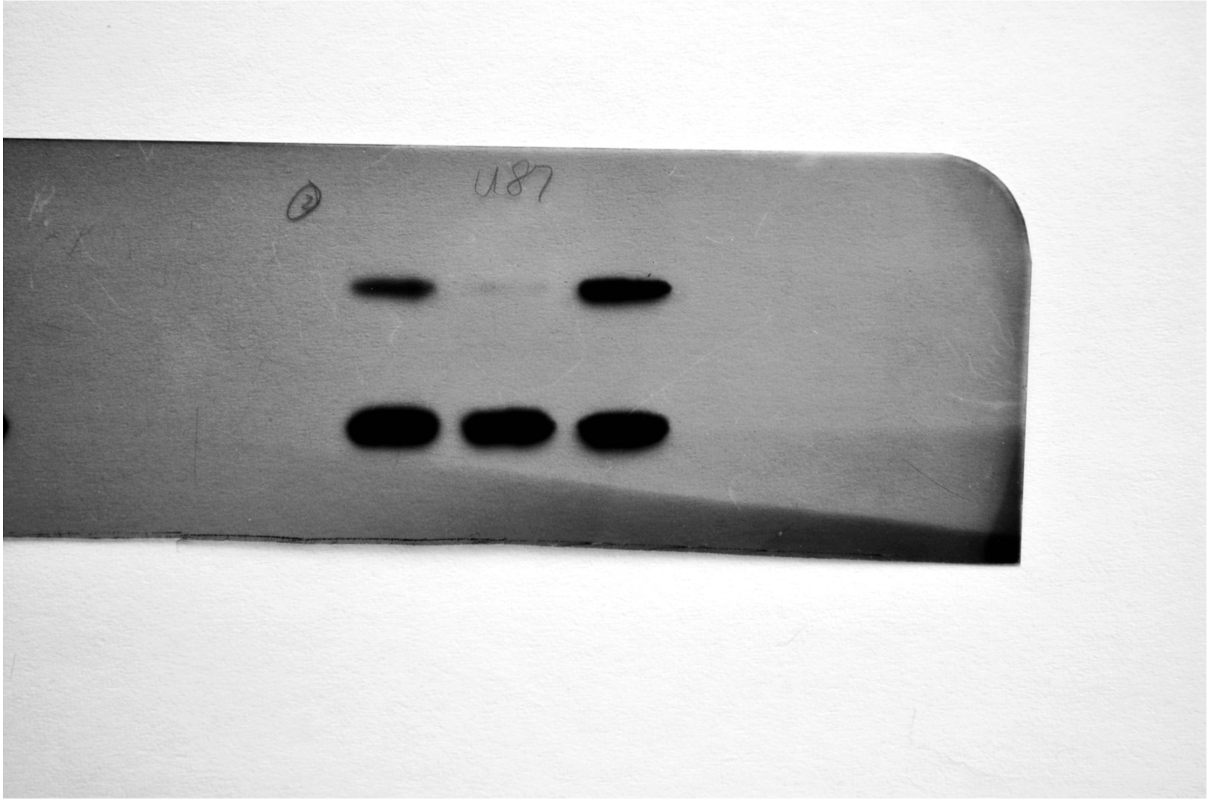

Figure 6a:

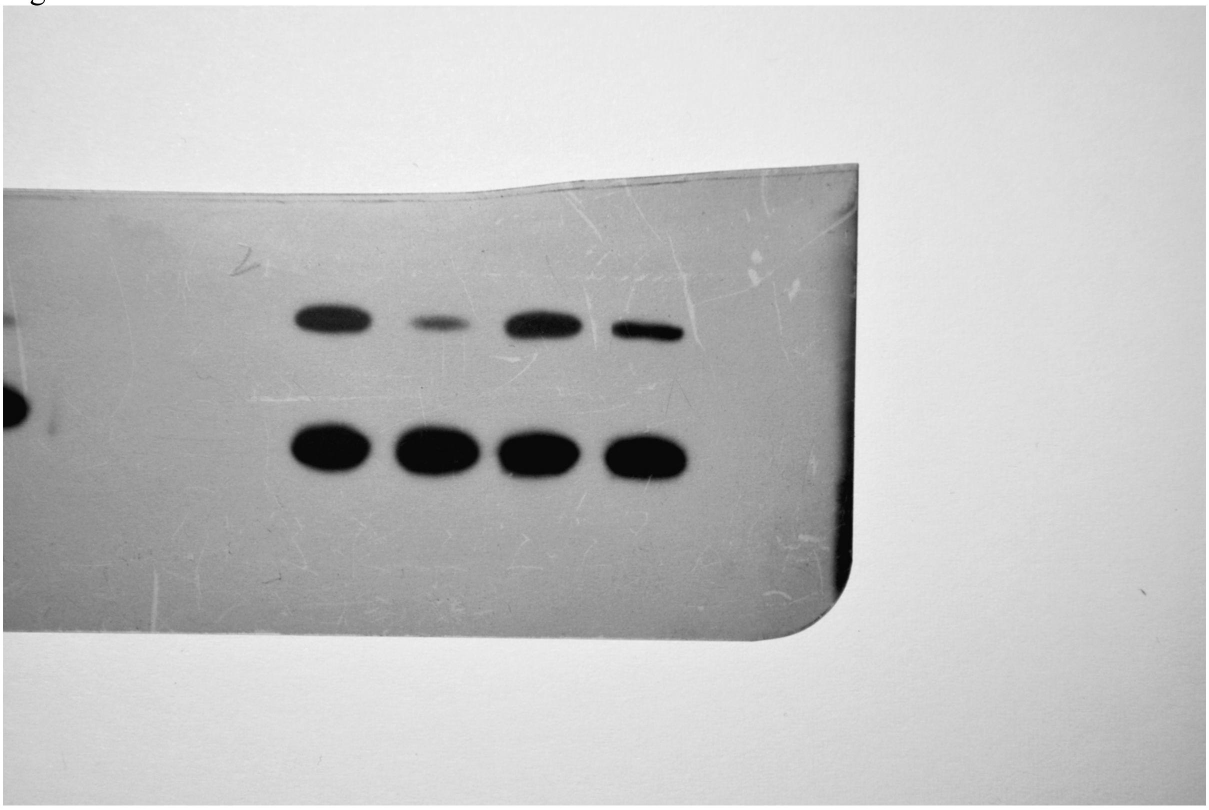

Figure: Differences in H&E Staining and GFAP Staining between Tumor Tissue and Peri-tumor Tissue

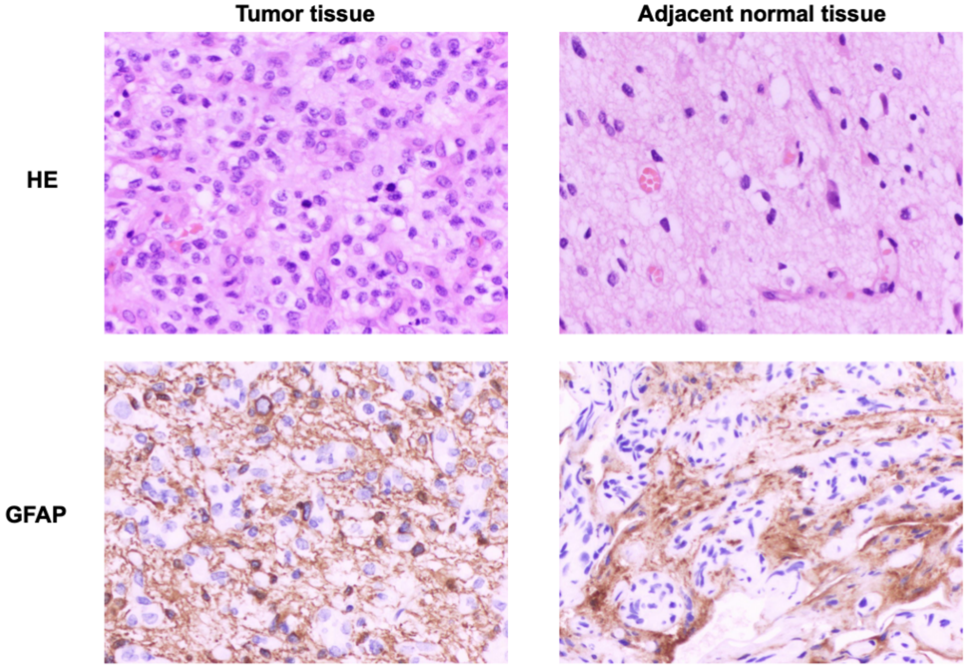

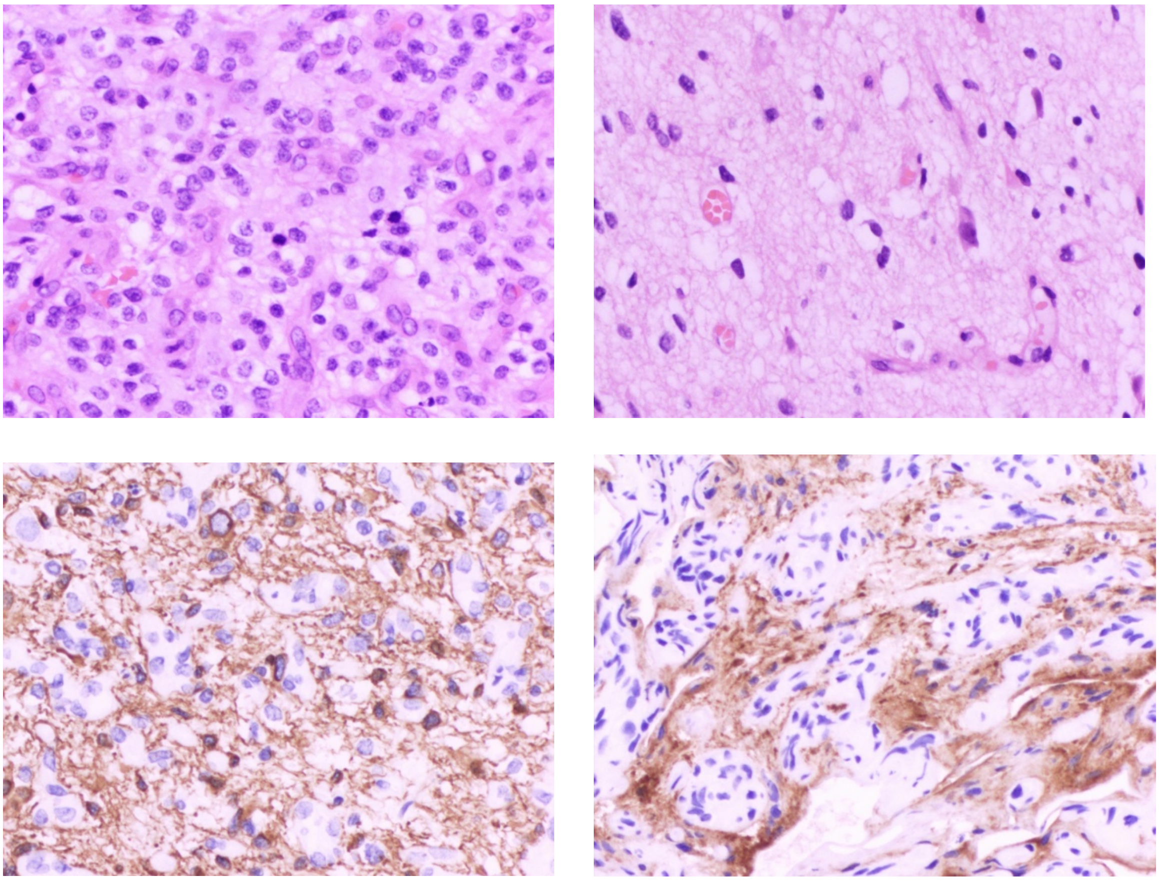

Supplement: Supplementary file 1 — Supplementary Information. [file 41598_2024_51976_MOESM1_ESM.pdf]
